# B=P double bonds relieved from steric encumbrance: matrix-isolation infrared spectroscopy of the phosphaborene F_2_B–P=BF and the triradical B=PF_3_†

**DOI:** 10.1039/d4sc01913j

**Published:** 2024-04-30

**Authors:** Mei Wen, Robert Medel, Pavel V. Zasimov, Carsten Müller, Sebastian Riedel

**Affiliations:** a Freie Universität Berlin, Institut für Chemie und Biochemie – Anorganische Chemie Fabeckstraße 34/36 14195 Berlin Germany s.riedel@fu-berlin.de

## Abstract

Free phosphaborenes have a labile boron–phosphorus double bond and therefore require extensive steric shielding by bulky substituents to prevent isomerisation and oligomerisation. In the present work, the small free phosphaborene F_2_B–P=BF was isolated by matrix-isolation techniques and was characterised by infrared spectroscopy in conjunction with quantum-chemical methods. In contrast to its sterically hindered relatives, this small phosphaborene exhibits an acute BPB angle of 83° at the CCSD(T) level. An alternative orbital structure for the B=P double bond is found in the triradical B=PF_3_, the direct adduct of laser-ablated atomic B and PF_3_. The single-bonded isomer F_2_B–PF and the dimer F_3_P–B≡B–PF_3_ are also tentatively assigned.

## Abstract 2

The B=PF_3_ triradical adduct is obtained by the reaction of laser-ablated boron atoms with PF_3_ in a solid neon matrix. Additionally, the phosphaborene F_2_B–P=BF without sterically demanding ligands but with an acute BPB angle is observed.

## Graphical abstract

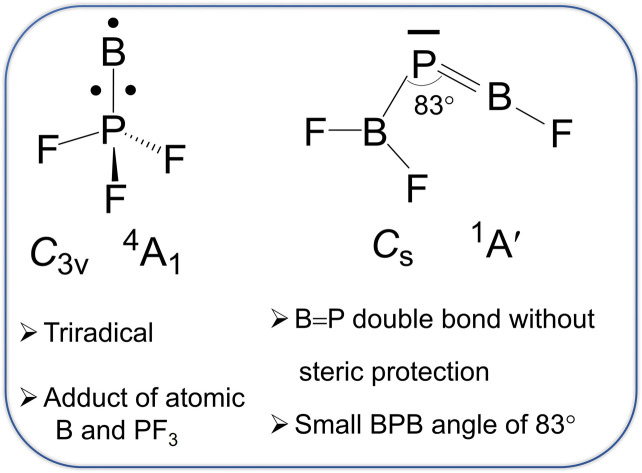

## Introduction

**Scheme 1:**
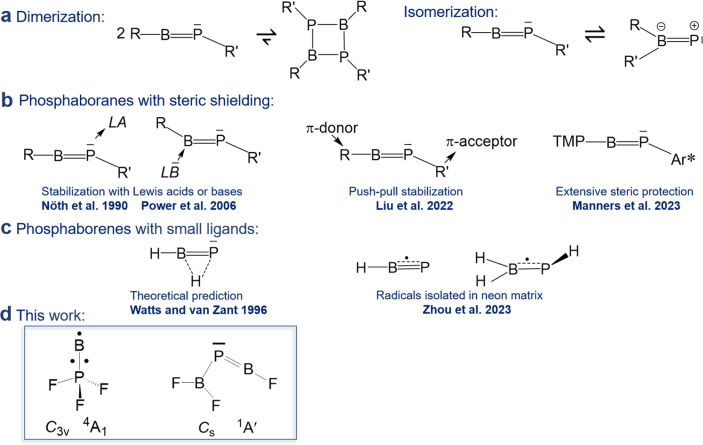
Overview of previous work on phosphaborenes. TMP = 2,2,6,6-tetramethyl piperidine, Ar* = 2,6-bis(triisopropylphenyl)-3,5-diisopropylphenyl.

Linear iminoboranes RB≡NR′ are isoelectronic to alkynes RC≡CR′ and subject of a maturing field or research.^1^ In contrast, reports on phosphaborenes RB=PR′ (also called boraphosphenes or phosphinidene boranes) were very rare for a longer period of time and only recently became more frequently. This backlog is due to the challenge of overcoming the very strong tendency of phosphaborenes to oligo- and isomerize (Scheme 1a), as they have a double bond rather than a triple bond, with a free electron pair on the phosphorus atom and a vacant p-orbital on the boron atom. This bonding situation is reflected in bent structures with a BPR′ angle that is strongly dependent on the substituents R and R′ in a range of at least 52–123°.^2,3^

The first free phosphaborene was observed in 1986 by mass spectrometry after thermolysis of its sterically encumbered dimer.^4^ The first room temperature stable phosphaborene was reported in 1990, obtained by ring cleavage, facilitated by additional coordination of a Lewis acid to the phosphorus atom for further steric shielding.^5^ As a counter-strategy, it was later shown that protecting the boron atom by coordinating a bulky Lewis base was also viable.^6–9^ Free phosphaborenes without further coordination could be isolated only very recently by utilizing substituents R and R′ that are either even more sterically demanding^10^ or combine π-acceptor and π-donor capabilities in a kind of push–pull cooperation, in addition to their bulkiness (Scheme 1b).^9^ However, extensive steric shielding also limits a possible application of phosphaborenes as further reagents.^10,11^ The design of substituents that balance stability and reactivity is therefore a current challenge.

Free phosphaborenes with sterically undemanding ligands were theoretically predicted to be thermodynamically and kinetically unstable, not only with respect to oligomerization, but also to 1,2-rearrangement to the RR′B=P isomer. Their experimental detection was therefore concluded to be unlikely.^3^ This also holds true for another theoretical investigation which considered further substitution patterns.^12^ However, a closer look at the calculations reveals that the picture is not as bleak as it was painted. For R = R′ = F, Cl or SiH_3_ the isomerization from RB=PR′ to RR′B=P was indeed calculated to be exergonic, but endergonic for R = R′ = H, OH or CH_3_.^3,12^ Despite this prediction, the parent phosphaborene HB=PH eluded experimental detection in recent work, but so did its isomers. Instead the radical species HBP and H_2_BPH were observed with formal B–P bond orders of 2.5 and 1.5, respectively (Scheme 1c).^13^ Furthermore, only homosubstituted (R = R′) small phosphaborenes have been considered in the above mentioned theoretical investigations, with the single exception of the ClB=PF species.^3,12^ On the contrary, it has recently been proposed that dissimilar ligands with opposite electronic properties (π-donor and π-acceptor abilities for R and R′, respectively) can be used to stabilize the RB=PR′ moiety.^9^

Indeed, in the present article we report the detection of by far the smallest free phosphaborene to date, F_2_B–P=BF (Scheme 1d), which exhibits this type of push–pull stabilisation (π-acceptor R′ = F_2_B and π-donor R = F). Other species with varying degrees of B=P double bonds have been assigned as well: the triradical B=PF_3_ and tentatively its isomer F_2_B–PF as well as its dimer F_3_P–B≡B–PF. These molecules have been prepared *via* the reaction of laser-ablated B atoms with PF_3_, isolated in solid neon matrices and characterized by FTIR spectroscopy and theoretical methods.

## Results and discussion

### Vibrational assignment

**Fig. 1:**
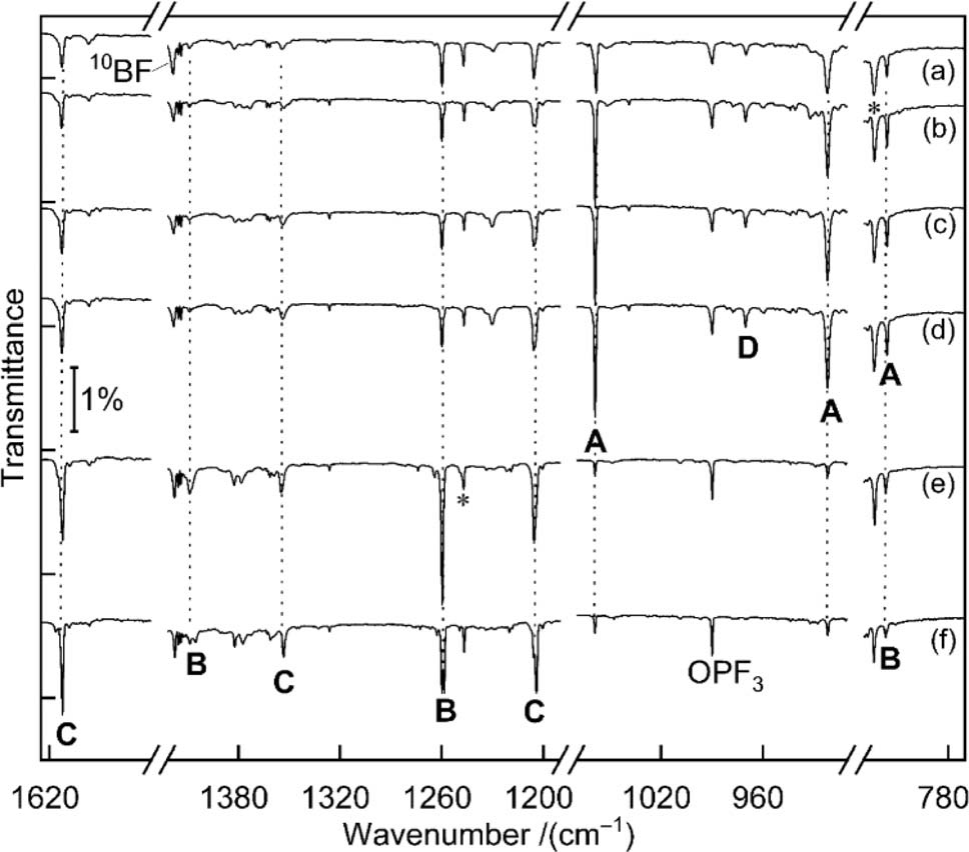
Infrared spectra obtained from codeposition of laser-ablated ^10^B-enriched boron atoms with 0.05% PF_3_ in solid neon matrices after (a) 60 min of sample deposition at 5 K, (b) 9 K annealing, (c) 10 min of 470 nm irradiation, (d) 10 min of 455 nm irradiation, (e) 15 min of > 220 nm irradiation, (f) 11 K annealing. A: B=PF_3_, B: F_2_B–PF (tentatively), C: F_2_B–P=BF, D: F_3_P–B≡B–PF_3_ (tentatively), unassigned bands are marked with asterisks.

Fig. 1 displays matrix-isolation FTIR spectra obtained after codeposition of laser-ablated ^10^B-enriched boron atoms with 0.05% PF_3_ in neon at 5 K. In addition to known absorptions from BF, BF_2_, BF_3_, PF_3_^−^, PF_5_ and OPF_3_,^14^ new product bands were observed.

#### B=PF_3_

A set of absorptions at 1058.9, 921.5 and 816.0 cm^−1^ (labeled as A) increased during sample annealing to 9 K and almost vanished upon subsequent irradiation (*λ* > 220 nm). This set is assigned to B=PF_3_, the direct addition product of the two reactants atomic B and PF_3_. Analogous experiments were performed with a boron target with isotopes in natural abundance (^10^B : ^11^B ≈ 1 : 4) (Fig. S1†). The 1058.9 cm^−1^ band exhibited a 19.8 cm^−1 10^B/^11^B isotopic shift and is assigned to the B=P stretching mode. The 921.5 cm^−1^ band experienced no detectable isotopic shift, indicative of the antisymmetric P–F stretching mode without boron participation. The weak band at 816.0 cm^−1^ showed a 13.4 cm^−1^ isotopic shift and is attributed to the breathing mode for the in-phase change of all four bond lengths. This assignment of B=PF_3_ is supported by the agreement with the calculated vibrational wavenumbers and their ^10^B/^11^B isotopic shifts at CCSD(T) and B3LYP levels of theory (Tables 1 and S1†).

**Table 1:** Observed (Ne matrices) and calculated (CCSD(T)/aug-cc-pVTZ level) stretching wavenumbers *ν* as well as ^10/11^B isotopic shifts Δ*ν* in cm^−1^ for B=PF_3_ (A), F_2_B–PF (B) and F_2_B–P=BF (C). IR intensities (in km mol^−1^) in parentheses were calculated at the B3LYP/aug-cc-pVTZ level

	Obs. *ν*(^10^B)	Cal. *ν*(^10^B)	Obs. Δ*ν* (^11^B)	Cal. Δ*ν* (^11^B)	Stretching mode
B=PF_3_ (*C*_3v_, ^4^A_1_)	816.0	813.9 (84)	13.4	14.1	Breathing
	921.5	921.7 (160 × 2)	0.0	0.0	Antis. PF_3_
	1058.9	1060.6 (219)	19.8	20.4	B=P
F_2_B–PF (*C*_s_, ^2^A′′)	—	637.3 (8)	—	5.2	B–P
	816.0	819.6 (114)	0.0	0.0	PF
	1260.0	1277.4 (323)	40.7	41.2	Sym. BF_2_
	1409.3	1454.8 (295)	45.3	50.5	Antis. BF_2_
F_2_B–P=BF (*C*_s_, ^1^A′)	—	628.6 (33)	—	5.3	B–P
	—	660.1 (2)	—	2.5	In-phase P=BF
	1205.4	1217.2 (569)	39.5	39.4	Sym. BF_2_
	1354.3	1384.5 (217)	45.6	47.4	Antis. BF_2_
	1613.1	1633.6 (548)	53.2–58.7 (resonance)	58.0	Out-of-phase P=BF

#### F_2_B–PF

A second set of absorptions (B) showed in contrast no growth on annealing but instead on irradiation. It is tentatively assigned to F_2_B–PF, a 1,2-rearrangement product of B=PF_3_. Two bands at 1260.0 and 1409.3 cm^−1^ are strongly shifted upon boron isotope substitution by 40.7 and 45.3 cm^−1^, they are attributed to the symmetric and the antisymmetric BF_2_ vibration modes, respectively. An expected weak band for the P–F stretching mode likely coincides with the 816.0 cm^−1^ band of set A that did not decline as sharply as expected from the change of the isotope ratio. This could be explained by the fact that only the breathing mode of B=PF_3_ is shifted by the change of boron mass while the pure P–F stretching mode of F_2_B–PF is not. Compared to the B=P stretching of B=PF_3_, the B–P stretching mode of F_2_B–PF is calculated to be substantially lower in wavenumber (1060.6 *vs.* 637.3 cm^−1^ at CCSD(T) level for ^10^B) and in IR intensity (219 *vs.* 9 km mol^−1^, calculated at B3LYP level), it is thus not observed. Because of the suspected overlap in the 816.0 cm^−1^ band and some deviation between the observed and calculated boron isotopic shift of the 1409.3 cm^−1^ band [50.5 *vs.* 45.3 cm^−1^, the latter value being consistent across different theoretical methods (Tables 1 and S1†)], we consider the assignment of F_2_B–PF as tentative.

#### F_2_B–P=BF

A third set of absorptions (C) also growths on irradiation but differently than set B, as apparent in the difference spectra upon selective 470 nm irradiation shown in Fig. S2.† Three bands at 1205.4, 1354.3 and 1613.1 cm^−1^ are assigned to F_2_B–P=BF, at least formally an insertion product of a second boron atom into the P–F bond of F_2_B–PF. The bands at 1205.4 and 1354.3 cm^−1^ of C show large ^11^B isotopic shifts of 39.5 and 45.6 cm^−1^ and are attributed to the symmetric and antisymmetric BF_2_ vibration modes of F_2_B–P=BF, respectively. The high wavenumber of the 1613.1 cm^−1^ band can be attributed to a strong out-of-phase coupling between the two bond stretches in the P=B–F moiety, amplified by its linear structure and the lightweight central boron atom. The corresponding in-phase combination is calculated as low as about 660 cm^−1^ but not observed due to negligible infrared activity. Isotopic substitution of boron leads to a large shift of the 1613.1 cm^−1^ band by 53.2–58.7 cm^−1^. A more accurate value cannot be determined because for the F_2_^10/11^B–P=^11^BF isotopologs the band is split into two components (1559.9 and 1554.4 cm^−1^) with similar intensities. A possible explanation might be a resonance between the out-of-phase P=^11^B–F stretching fundamental (calculated in the harmonic approximation at 1575.6 cm^−1^ for F_2_^11^B–P=^11^BF) and the combination mode (estimated at 1567.2 cm^−1^) of two quanta of P=^11^B–F bending (2 × 454.8 cm^−1^) and one quantum of in-phase P=^11^B–F stretching (657.6 cm^−1^) with matching symmetry (*a*′) and spatial location. For F_2_^10^B–P=^10^BF the calculated difference (1633.6 *vs.* 1601.1 cm^−1^) is larger, explaining the absence of the suspected resonance. Because all modes are largely localized at either side of the phosphorous atom, the vibrational wavenumbers of the two mixed ^10/11^B isotopologs are calculated to coincide within 1 cm^−1^ with the corresponding ones of the isotopically pure species (Table S2†), in line with the absence of any further resolved spectral splitting.

#### F_3_P–B≡B–PF_3_

The band at 970.0 cm^−1^ in the ^10^B-enriched experiment (labeled as D in Fig. 1) increased during sample annealing to 9 K and almost vanished upon irradiation with *λ* > 220 nm.

In the natural abundance boron experiments the band splits into three absorptions at 970.0, 960.4 and 952.5 cm^−1^ with approximately 1 : 8 : 16 relative intensities (Fig. S3†), which indicates that the observed species features two equivalent boron atoms. Based on the comparison with calculated band positions and isotopic shifts (Table S3†) this band is tentatively assigned to the antisymmetric B–P stretching mode of F_3_P–B≡B–PF_3_. This species is likely be formed by the dimerization of the triradical B=PF_3_, analogous to OC–B≡B–CO from B=CO.^15^ However, B(PF_3_)_2_ was not observed, in contrast to its analogue B(CO)_2_. For F_3_P–B≡B–PF_3_ only one other band with significant IR activity is predicted (Table S4†) in the detector range (>450 cm^−1^). However, it could not be identified, potentially due a combination of its expected weak intensity and spectral overlap. We therefore suggest to consider the assignment of F_3_P–B≡B–PF_3_ as tentative.

### Theoretical characterizations

#### B=PF3

**Fig. 2:**
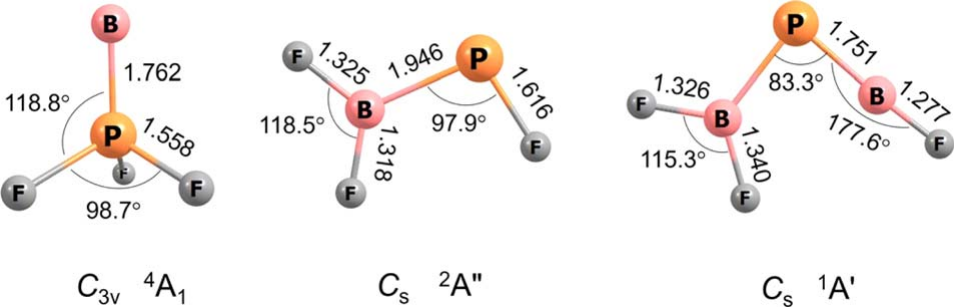
Optimized structures at the CCSD(T)/aug-cc-pVTZ level of theory. Bond lengths (Å), bond angles (°) and molecular symmetries are also shown.

For B=PF_3_ two electronic states, ^4^A_1_ with *C*_3v_ symmetry and ^2^A′ with *C*_s_ symmetry, are calculated to be very similar in energy, the former higher at CCSD(T)/aug-cc-pVTZ level by 1.6 kcal mol^−1^. Curiously, the observed band positions agree only with the higher energy ^4^A_1_ state. However, this energy difference almost vanishes with aug-cc-pVQZ single-point correction and reverses in sign with aug-cc-pV5Z (Table S5†). The calculated properties and optimised structures of the unobserved 2A′ state are shown in the ESI in Table S6 and Fig. S7,† respectively. CCSD(T) calculations predict a B–P bond length of 1.762 Å for the assigned ^4^A_1_ state (Fig. 2). This value is significantly shorter than reported B–P single bond lengths (1.92–2.00 Å) and at the lower end of B=P double bond lengths (1.763–1.853 Å).^6,7,16^ The Wiberg bond index (WBI) of 1.472 (calculated at B3LYP level) as well indicates at least a partial double bond character.

**Fig. 3:**
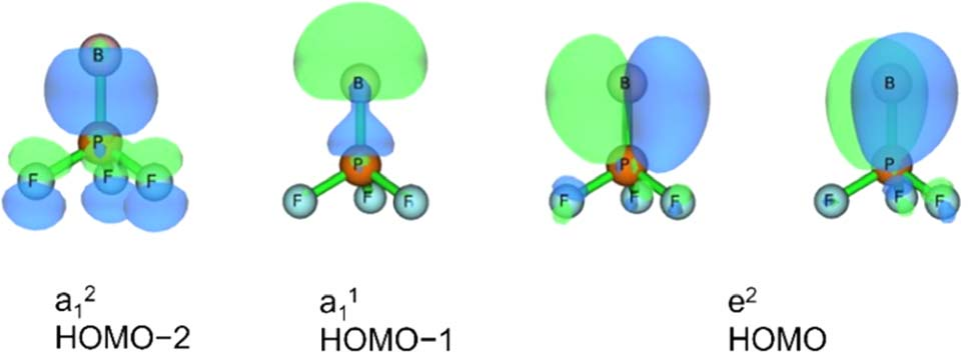
Molecular orbitals of B=PF3 (*C*_3v_, ^4^A_1_): HOMO–2, HOMO–1 and HOMO with symmetries and occupation numbers, calculated at HF/aug-cc-pVTZ//CCSD(T)/aug-cc-pVTZ level.

Three unpaired electrons are distributed over a doubly degenerate B–P π-bonding HOMO with largely boron p character and a non-bonding HOMO–1 of largely boron sp character, see Fig. 3. In addition, there is a doubly occupied σ-bonding HOMO–2 for an overall B–P formal bond order of two. The total spin density is predominantly located at the boron atom (Fig. S5†). In order to further understand the interaction between B and PF_3_, the charge flow upon combination of the B and PF_3_ fragments were visualized with ETS-NOCV (Extended Transition State-Natural Orbital for Chemical Valence) calculations,^17^ deformation maps are shown in Fig. S4.† Dative character of the PF_3_→B σ-bonding and π-backdonation is indicated. This bonding situation of B=PF_3_ is analogous to the known ^4^Σ^−^ B=CO.^18^

#### F_3_P–B≡B–PF_3_

The tentatively assigned F_3_P–B≡B–PF_3_ is predicted to show a ^1^A_1g_ ground state in *D*_3d_ symmetry at B3LYP/aug-cc-pVTZ level. The optimized structure (Fig. S6†) shows a very short B–B bond (1.430 Å, WBI = 2.37), even shorter than the one in O≡C–B≡B–C≡O (1.444 Å, WBI = 1.97 calculated at the same level of theory).^15^ The formal B≡B triple bond is derived from a σ-bonding orbital (HOMO–1) and a doubly degenerate π-bonding orbital (HOMO) (Fig. S12†). Based on the strong π-acceptor properties of PF_3_, the HOMO is substantially delocalized over the B–P bonds, so that F_3_P–B≡B–PF_3_ retains some of the B=P double-bond character (1.768 Å, WBI = 1.19) of its monomer B=PF_3_ (1.757 Å, WBI = 1.55 at the same level of theory). This is also reflected in the natural resonance theory (NRT) analysis, where, besides structures with the leading F_3_P–B≡B–PF_3_ motif (48%), also resonance forms that show one or two B=P double bonds have significant combined weights of 14% and 20%, respectively (Fig. S13†). The spin-allowed dimerization energy of 2 B=PF_3_ (^4^A_1_) → F_3_P–B≡B–PF_3_ (^1^A_1g_) is computed to be −152.6 kcal mol^−1^.

#### F_2_B–PF

F_2_B–PF is predicted to have a ^2^A′′ ground state with *C*_s_ symmetry, in contrast to its hydrogen analogue H_2_B–PH that shows a non-planar structure.^13^ F_2_B–PF is computed to be the most stable isomer of its formula, 111.3 kcal mol^−1^ below the doublet B=PF_3_ compound. As shown in Fig. S5,† the unpaired electron is located in a HOMO with dominant phosphorus p_*z*_ character with only very minor contribution from the boron atom. The B–P bond order is therefore only very slightly higher than one, which is reflected in a WBI of 1.122. This is in contrast to the trifluorovinyl radical F_2_C=CF which is instead a σ radical with a double bond,^19^ further emphasizing the differences between carbon–carbon and boron–phosphorus compounds.

#### F_2_B–P=BF

F_2_B–P=BF can be characterized as a free phosphaborene RB=PR′ with small substituents (R = F and R′ = BF_2_). The observation of a member of this class of molecules is somewhat unexpected, as it was thought to be thermodynamically and kinetically unstable with respect to spontaneous unimolecular rearrangement in 1,2-shift reaction.^3^ Yet, F_2_B–P=BF is calculated to be the most stable isomer, 31.0 kcal mol^−1^ lower in energy than the second lowest isomer (F_2_B)FB=P at B3LYP/aug-cc-pVTZ level. This might be explained by the recently proposed push–pull stabilization of the B=P double bond by a π-donor (–F) and a π-acceptor (–BF_2_) which diminish the Lewis acidity and basicity of boron and phosphorous atoms, respectively.^9^ The optimized structures and relative energies of all obtained isomers of the formula B_2_PF_3_ are provided in Fig. S11.† However, in the absence of bulky ligands, the dimerization of F_2_B–P=BF is still computed to be considerably exothermic by −49.3 kcal mol^−1^ at B3LYP level. However, its dimerization is prevented under matrix-isolation conditions.

**Fig. 4:**
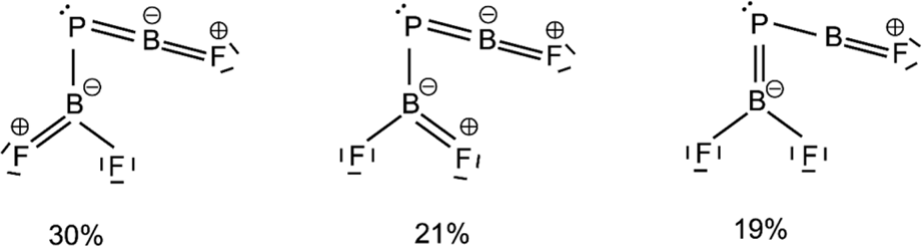
Leading resonance structures for F_2_B–P=BF from natural resonance theory (NRT). Further structures with weights of less than 8% are available in Fig. S8.†

The B=P and P–B bond distances are computed to be 1.751 and 1.925 Å (Fig. 2) with WBIs of 1.866 and 1.073, respectively. Bond orders of two and one are also present in the dominant resonance structures from the NRT analysis (Fig. 4). Other NRT structures with lower weights are shown in Fig. S8.† NRT resonance structures with a B≡P triple bond have a combined weight of 21%, those with a double bond of 55% and those with a single bond of 22%. The B–F bond length to the singular fluorine ligand is very short (1.277 A) at CCSD(T) level, if comparable with the diatomic BF molecule (calculated 1.275 Å, experimental 1.263 Å (ref. 20)) which can be explained by boron sp-hybridization and two B ← F π-donation interactions, both in and orthogonal to the molecular plane. In contrast, the B–F bonds in the BF_2_ moiety are slightly elongated (1.326 and 1.340 Å) when compared to both F_2_B–PF (1.325 and 1.318 Å) and BF_3_ (calculated 1.315 Å, experimental 1.311 Å (ref. 21)), which is likely due to competition between B ← F and B ← P π-donation. In line with an increase in B ← P π-donation, the P–B bond length in F_2_B–P=BF (1.925 Å) is slightly shorter than in F_2_B–PF (1.946 Å).

**Fig. 5:**
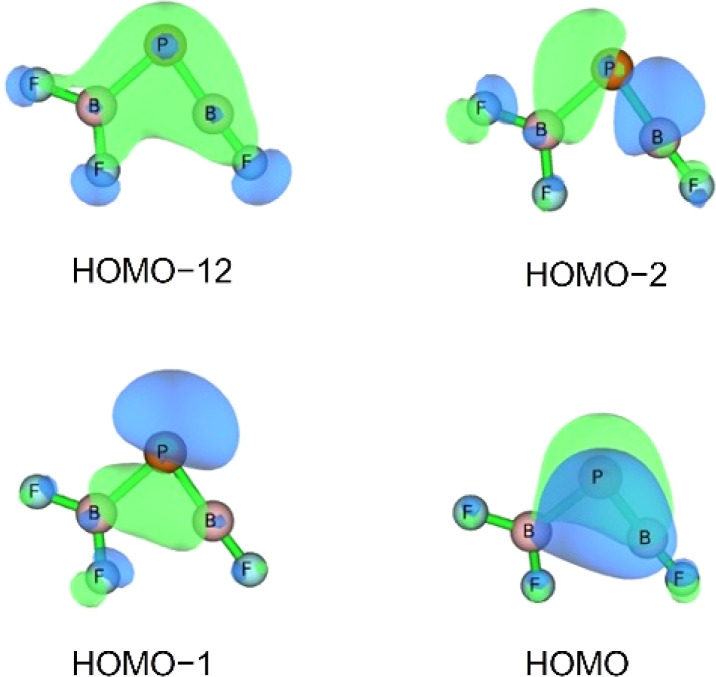
Selected bonding molecular orbitals of F_2_B–P=BF calculated at HF/aug-cc-pVTZ//CCSD(T)/aug-cc-pVTZ level.

The HOMO of this compound is a polarized π-bonding orbital with leading contributions from the phosphorus atom (64% according to Hirshfeld partition), the two-coordinated boron atom (22%) and the tri-coordinated boron atom (9%). Despite this moderate 3-center-2-electron bonding character, as previously reported for a similar free phosphaborene BPB moiety,^9^ the B=P and P–B interactions clearly have very different overall bond orders. The HOMO–1 resembles mostly an in-plane lone pair orbital of P (71% contribution) which contributes little to B=P and P–B bonding according to Mulliken and Mayer bond order decomposition (Tables S8–S11†).^22^ This is more consistent with the lone pair interpretation for previously isolated free phosphaborenes,^10^ but less consistent with the bonding picture presented by Su *et al.* who propose a second in-plane π-bond for an overall triple bond.^3^ Nevertheless, the HOMO–1 shows a boron contributions [12% B(F_2_), 10% B(F)] that seem to be of some significance for the structure of the molecule. A striking feature of F_2_B–P=BF is the acute BPB bond angle of 83°, which cannot be explained by VSEPR or hybridisation considerations alone. In contrast, the BPR′ bond angles of previously experimentally detected free phosphaborenes are obtuse and amount to 115.5(1)^10^ and 106.00(6),^9^ which are most likely broadened by steric repulsion between the bulky substituents. Conversely, the acute angle in F_2_B–P=BF might suggest an attractive interaction. Indeed, the WBI of 0.156 for the B⋯B interaction at 2.45 Å is small but not negligible, as are other evaluated bond order measures (Table S7†). A small minority of NRT structures even feature a covalent B–B single bond with a low combined weight of 2% (Fig. S8†). It is tempting to link this attraction to the three-center character of the π-type HOMO, but Mulliken and Mayer bond order decomposition instead suggests leading contributions from in-plane orbitals, such as HOMO–1 and HOMO–12 (Tables S12 and S13†). While one orbital lobe of HOMO–1 resembles a phosphorus lone pair, the other connects the two boron nuclei (Fig. 5). This interpretation is further supported by decomposition of the B⋯B Wiberg bond index in NAO basis, with about 2/3 being contributed by interactions involving the in-plane p_*x*_ and p_*y*_-orbitals and 1/3 by those involving the out-of-plane p_*z*_-orbitals of the boron atoms (Table S14†). There is no (3, −1) critical point located between the boron nuclei according to the quantum theory of atoms in molecules (QTAIM). An overall BPB multi-center bond order in NAO basis of 0.16 is calculated, somewhat lower than the values for the textbook examples of the allyl cation (0.22) and diborane (0.25) calculated at the same level of theory.

To explore whether this feature is inherent to F_2_B–P=BF or more widespread, we optimized at CCSD(T) level the structure of a number of other small phosphaborenes. The isomer with swapped substituents, F_2_B–B=PF, is computed to be less stable by 50.9 kcal mol^−1^ and its BPF angle amounts to 102°. Similar obtuse BPR′ angles are obtained for FB=PF (99°) and FB=PCH_3_ (97°). In contrast, acute angles are found for FB=PH (80°) and FB=PSiH_3_ (79°). Small angles correlate with higher B⋯R′ WBIs and BPR′ multi-center bond orders (Fig. S9†), although there are clearly other contributing factors, such as the size of the ligand R′. Extreme cases are the non-classical structures of the parent phosphaborene HB=PH,^2^ in which one hydrogen atom effectively bridges the B=P bond (53°), and the hydrogen homolog of F_2_B–P=BF: B_2_PH_3_ (56°). The latter converges to a structure with *C*_2v_ symmetry, with one hydrogen atom and the phosphorus atom bridging either side of the B–B bond with a distance of 1.70 Å (Fig. S10†). Clearly, a more systematic study of substitution effects on the unusual bonding situations and stability of small phosphaborenes is required.

### Formation pathways

**Scheme 2:**
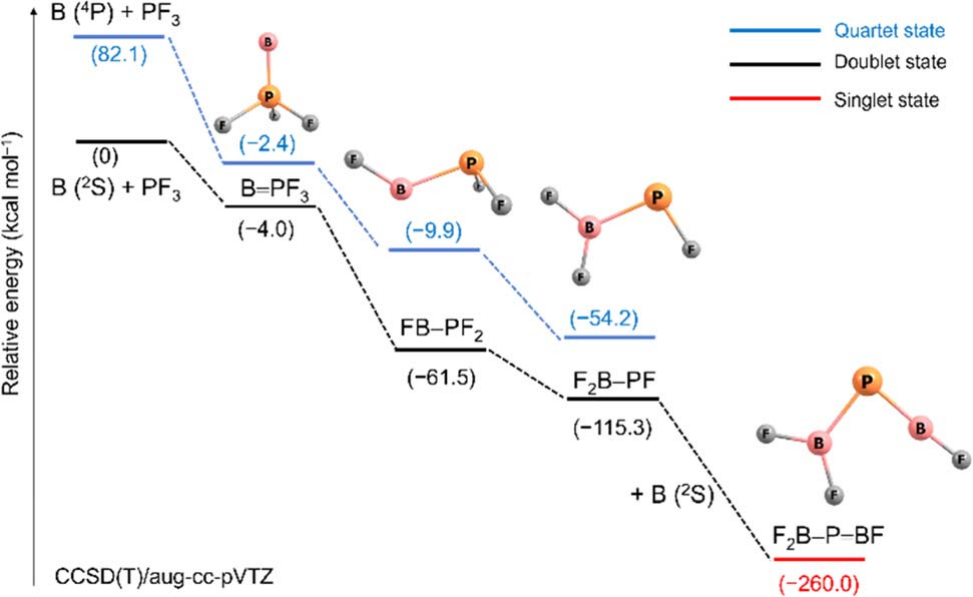
Relative stabilities (electronic energies + ZPE correction) in kcal mol^−1^ for species formed from laser ablated boron atoms with PF_3_ at the CCSD(T)/aug-cc-pVTZ level (distances not to scale).

Scheme 2 outlines relative energies and possible pathways for the formation of the observed species. Because overall spin multiplicity is conserved, a plausible route for the formation of the observed quartet B=PF_3_ is the reaction of singlet PF_3_ with a laser-ablated boron atom in an excited ^4^P (2s^1^2p^2^) state^23^ (2s → 2p promotion, 82.1 kcal mol^−1^ excitation energy). However, the apparent growth of B=PF_3_ on annealing suggests that it can be formed as well in cryogenic matrices without activation energy. Indeed, the formation of B=PF_3_ in its excited doublet state by reaction of PF_3_ with a ground state ^2^P (2s^2^2p^1^) boron atom is calculated to be slightly exothermic (−4.0 kcal mol^−1^). The subsequent fast intersystem crossing (ISC) to the quartet state could explain the non-observation of the doublet state. Triggered by irradiation, exothermic stepwise 1,2-rearrangement would then lead first to the unobserved intermediate FB–PF_2_ (−59.1 kcal mol^−1^), quickly followed by further isomerization to the most stable isomer F_2_B–PF (−53.8 kcal mol^−1^). Transition states and barriers are reported at B3LYP level in the ESI.† Finally, the phosphaborene F_2_B–P=BF could be produced by irradiation-induced insertion of a second boron atom in the remaining P–F bond of F_2_B–PF. This step is calculated to be exothermic by −144.7 kcal mol^−1^ which is by far more exothermic than the insertion of a B atom into one of the P–F bonds of PF_3_ (−61.5 kcal mol^−1^). This sequence of initial barrier-free B atom adduction, light-induced isomerisation and second B atom insertion appears to be the most plausible pathway. The formation of F_2_B–P=BF by reaction of B_2_ with PF_3_ was also considered but none of the expected intermediates (Scheme S2†) were observed.

## Conclusions

In summary, the reaction of laser-ablated atomic boron and phosphorus trifluoride produced a variety of previously unreported boron–phosphorus compounds that were characterized by matrix-isolation infrared spectroscopy and quantum-chemical methods. B=PF_3_ and F_2_B–P=BF both feature a boron–phosphorus double bond but with different orbital structures. The latter molecule is a phosphaborene without protection from sterically demanding substituents, a class of molecules previously thought to be too unstable to be observed experimentally.

Furthermore, we tentatively assign two new species, the single bonded F_2_B–PF and the dimer F_3_P–B≡B–PF_3_ with a delocalised π-system. We hope that these findings will contribute to molecular design in the emerging field of multiple-bonded boron–phosphorus compounds.

## Author contributions

M. W. planed and performed the experiments, carried out the quantum-chemical calculations and wrote the first draft of the manuscript. R. M. performed some theoretical calculations and bonding analysis, and revised the manuscript. P. Z. analyzed the experiment result. C. M. revised the manuscript. S. R. guided and advised the project and proofread the manuscript.

## Conflicts of interest

There are no conflicts to declare.

## Supplementary Material

SC-015-D4SC01913J-s001
