# Supplementary material for: B[double bond, length as m-dash]P double bonds relieved from steric encumbrance: matrix-isolation infrared spectroscopy of the phosphaborene F2B–P[double bond, length as m-dash]BF and the triradical B[double bond, length as m-dash]PF3
Source: Chem Sci. 2024 Apr 30;15(21):8045–51. doi: 10.1039/d4sc01913j (PMC11134404; doi:10.1039/d4sc01913j)
Supplement: SC-015-D4SC01913J-s001 [file SC-015-D4SC01913J-s001.pdf]

Electronic Supplementary Material (ESI) for Chemical Science.
This journal is © The Royal Society of Chemistry 2024

Supporting Information

B=P Double Bonds Relieved from Steric Encumbrance: Matrix-Isolation Infrared Spectroscopy of the Phosphaborene $F_2B{-}P{=}BF$ and the Triradical $B{=}PF_3$

Mei Wen,[a] Robert Medel,[a] Pavel Zasimov,[a] Carsten Müller,[a] Sebastian Riedel*[a]

[a] M. Sc. Mei Wen, Dr. Robert Medel, Dr. Pavel Zasimov, Dr. Carsten Müller, and Prof. Dr. Sebastian Riedel,
Freie Universität Berlin, Institut für Chemie und Biochemie–Anorganische Chemie, Fabeckstrasse 34/36, 14195 Berlin, Germany. E-Mail: s.riedel@fu-berlin.de

Contents

Experimental methods..........4
Theoretical methods..........4
Figure S1. IR spectra in neon matrix of natural boron atoms and isotopic ^{10}-boron atoms reacted with PF_3..........5
Figure S2. IR spectra in neon matrix of natural boron atoms and isotopic ^{10}-boron atoms reacted with PF_3..........6
Figure S3. IR spectra in neon matrix of natural boron atoms and isotopic ^{10}-boron atoms reacted with PF_3..........7
Figure S4. ETS-NOCV deformation maps for B=PF_3 (C_{3v}, $^4A_1$)..........8
Figure S5. Spin density of B=PF_3 (C_{3v}, $^4A_1$) and F_2B–PF..........8
Table S1. Calculated stretching wavenumbers ν (in cm^{-1}) and $^{10/11}$B isotopic shifts of compounds **A**, **B** and **C** at the DFT level..........9
Table S2. Calculated stretching wavenumbers ν (in cm^{-1}) of compound **C** F_2B–P=BF..........9
Table S3. Observed and calculated stretching wavenumbers ν (in cm^{-1}) and $^{10/11}$B isotopic shifts (Δν, cm^{-1}) of F_3P–B≡B–PF_3..........9
Table S4. All calculated stretching wavenumbers ν (in cm^{-1}) of F_3P–^{11}B≡^{11}B–PF_3 at the B3LYP/aug-cc-pVTZ level..........10
Table S5. The energies E in (a.u.) and the difference energies ΔE in (kcal mol^{-1}) for a given basis set for the $^4A_1$ and $^2A'$ states of BPF_3..........10
Figure S6. Optimized geometry of dimer F_3P–^{11}B≡^{11}B–PF_3 at the B3LYP/aug-cc-pVTZ level of theory..........10
Table S6. Calculated stretching wavenumbers ν (in cm^{-1}) and $^{10/11}$B isotopic shifts (Δν, cm^{-1}) of B–PF_3(C_s, $^2A'$)..........11
Figure S7. Optimized geometries of BPF_3 (C_s, $^2A'$) compound at the CCSD(T)/aug-cc-pVTZ level of theory..........11
Table S7. Selected calculated properties of F_2B–P=BF at CCSD(T)/aug-cc-pVTZ level..........11
Table S8. All occupied orbitals contribution to Mulliken bond order for the B=P bond of F_2B–P=BF at CCSD(T)/aug-cc-pVTZ level..........12
Table S9. All orbital occupancy-perturbed Mayer bond order[1]for the B=P bond of F_2B–P=BF at CCSD(T)/aug-cc- pVTZ level..........13
Table S10. All occupied orbitals contribution to Mulliken bond order for the P–B bond of F_2B–P=BF at CCSD(T)/aug-cc-pVTZ level..........14
Table S11. All orbital occupancy-perturbed Mayer bond order[1] for the P–B bond of F_2B–P=BF at CCSD(T)/aug-cc-pVTZ level..........15
Table S12. All occupied orbitals contribution to Mulliken bond order for the B···B interaction in F_2B–P=BF at CCSD(T)/aug-cc-pVTZ level..........16
Table S13. All orbital occupancy-perturbed Mayer bond order[1] for the B···B interaction in F_2B–P=BF at CCSD(T)/aug-cc-pVTZ level..........17
Table S14. Wiberg bond order decomposition in NAO basis for the B···B interaction in F_2B–P=BF at CCSD(T)/aug-cc-pVTZ level..........18

Figure.S8 Four predominant resonance structures (weight < 8%) and their weights for the compound $F_2B–P=BF$ at the B3LYP/aug-cc-pVTZ level of theory………………19
Figure S9. Calculated properties for different small phosphaborenes RB=PR' at CCSD(T)/aug-cc-pVTZ level………..…………………………………….……..19
Figure S10. Optimized structures for different small phosphaborenes RB=PR' at the CCSD(T)/aug-cc-pVTZ level…………..……………………………………………20
Figure S11. Isomers of compound C with relative energies in kcal mol^{-1} calculated at B3LYP/aug-cc-pVTZ level of theory……………………………………………..20
Figure S12. Selected bonding molecular orbitals of dimer $F_3P–B≡B–PF_3$ calculated at B3LYP/aug-cc-pVTZ level of theory……………………………………………..21
Figure S13. Leading resonance structures for dimer $F_3P–B≡B–PF_3$ from natural resonance theory (NRT)……………………………………………………………..21
Scheme S1. Reaction mechanism for laser ablated boron atoms with PF_3 at the B3LYP/aug-cc-pVTZ
level……………………………………………………...22
Figure.S14. Transition state structure (TS1-D) (^{2}A, C_1) and its frequencies..……….22
Figure.S15. Intrinsic reaction coordinate (IRC) via the transition state (TS1-D) (^{2}A, C_1) for the mutual isomerization.……………………………………………………23
Figure.S16. Transition state structure (TS1-Q) (^{4}A", C_S) and its frequencies..………23
Figure.S17. Intrinsic reaction coordinate (IRC) via the transition state (TS1-Q) (^{4}A", C_S) for the mutual isomerization.…………………………………………………..24
Figure.S18. Transition state structure (TS2-D) (^{2}A, C_1) and its frequencies..……….24
Figure.S19. Intrinsic reaction coordinate (IRC) via the transition state (TS2-D) (^{2}A, C_1) for the mutual isomerization.……………………………………………………25
Figure.S20. Transition state structure (TS2-Q) (^{4}A, C_1) and its frequencies..……..…25
Figure.S21. Intrinsic reaction coordinate (IRC) via the transition state (TS2-Q) (^{4}A, C_1) for the mutual isomerization.……………………………………………………26
Scheme S2. Reaction mechanism for laser ablated two boron atoms with PF_3 at the B3LYP/aug-cc-pVTZ
level……………………………………………………...26
Calculated atomic coordinates of compounds **A, B, C, D**, transition states and small phosphaborenes RB=PR'.……………………..…………………………………27
References……………………………………………………………………..31

Experimental methods

The experimental setup for matrix isolation infrared spectroscopy has been described in more detail in our previous works.[2] Briefly, the 1064 nm fundamental of a Nd:YAG laser (Continuum, Minilite II, 10 Hz repetition rate) with energy of 50–65 mJ per 10 ns pulse was used to ablate a rotating bulk boron target to produce boron atoms. Natural abundance boron (^{10}B, 19.8%; ^{11}B, 80.2%) or ^{10}B-enriched (> 95%) targets were used. The ablated boron atoms were co-deposited with 0.05% PF_3 (99%) in neon inside a high vacuum chamber onto a gold-plated copper mirror cooled to 5 K by using a closed-cycle helium cryostat (Sumitomo Heavy Industries, RDK-205D). FTIR spectra were recorded with a Bruker Vertex 80v spectrometer at 0.5 cm^{-1} resolution in the region between 4000 and 450 cm^{-1} using a liquid nitrogen cooled mercury cadmium telluride (MCT) detector. The matrix samples were irradiated with a 470 nm LED (Intelligent LED Solutions ILH-ON04-BLUE-SC211-WIR200) or a mercury arc lamp (Osram HQL 250, 175 W) with the outer globe removed.

Theoretical methods

Structures and harmonic frequencies calculated with density functional theory (DFT) were performed using the Gaussian 16 program version A.03 package[3] employing the hybrid functional B3LYP.[4] The further high level coupled-cluster singles-doubles with perturbational triples excitations (CCSD(T))[5] calculations were carried out in the Molpro 2019.1.0. software package.[6] For all calculations, Dunning's correlation consistent polarized basis sets of triple-zeta quality with diffuse augmentation functions (aug-cc-pVTZ)[7] were used. The further wave-function analysis were carried out by NBO 7.0[8] and Multiwfn Version 3.8 (dev).[9] The latter software was also used to visualize the molecular orbitals. Molecular structures were visualized with the program Chemcraft version 1.8.[10]

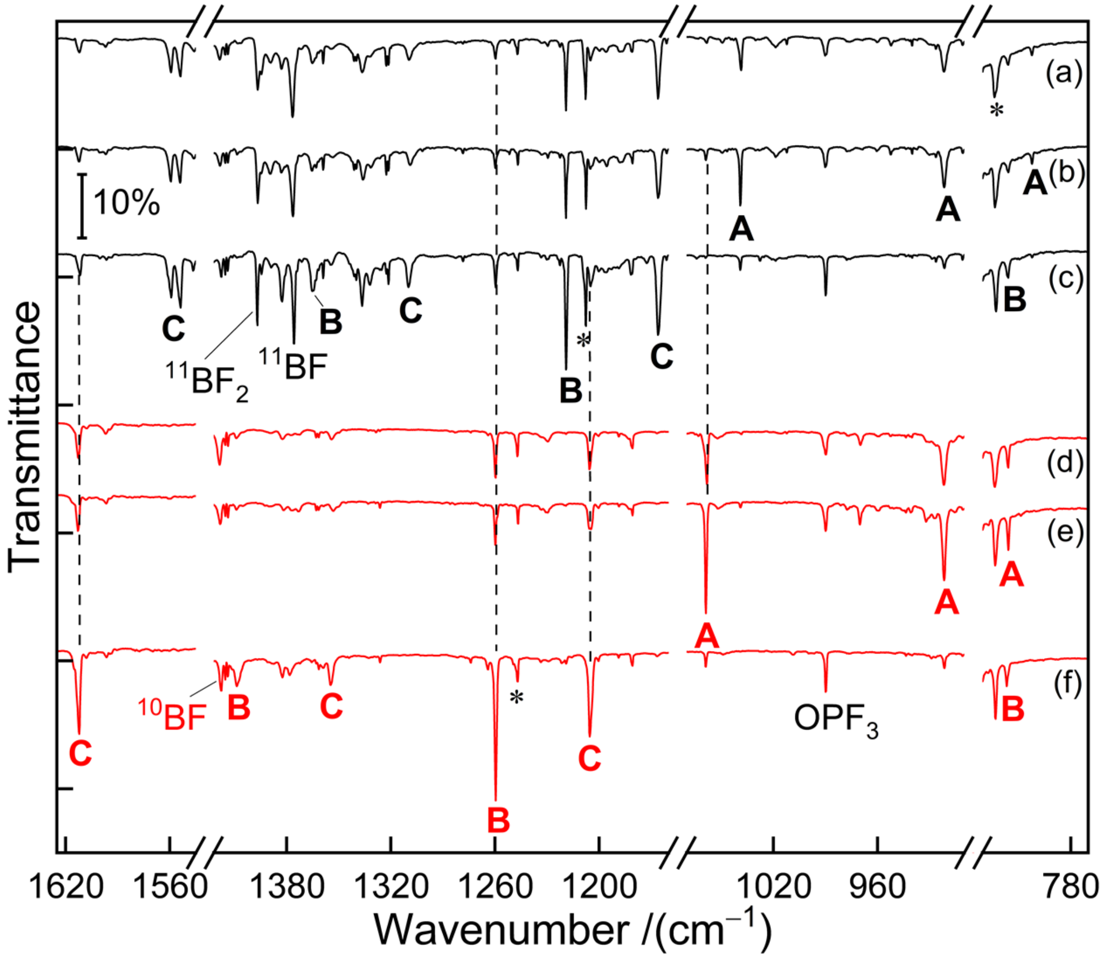

Figure S1. Infrared spectra obtained from codeposition of laser-ablated boron atoms in natural-abundance (a, b, c) and ^{10}B-enriched (d, e, f) with 0.05% PF_3 in neon (a, d), annealing to 9 K (b, e), and 15 min of $\lambda > 220$ nm irradiation (c, f). **A**: $B{=}PF_3$, **B**: $F_2B{-}PF$, **C**: $F_2B{-}P{=}BF$, unassigned bands are marked with asterisks.

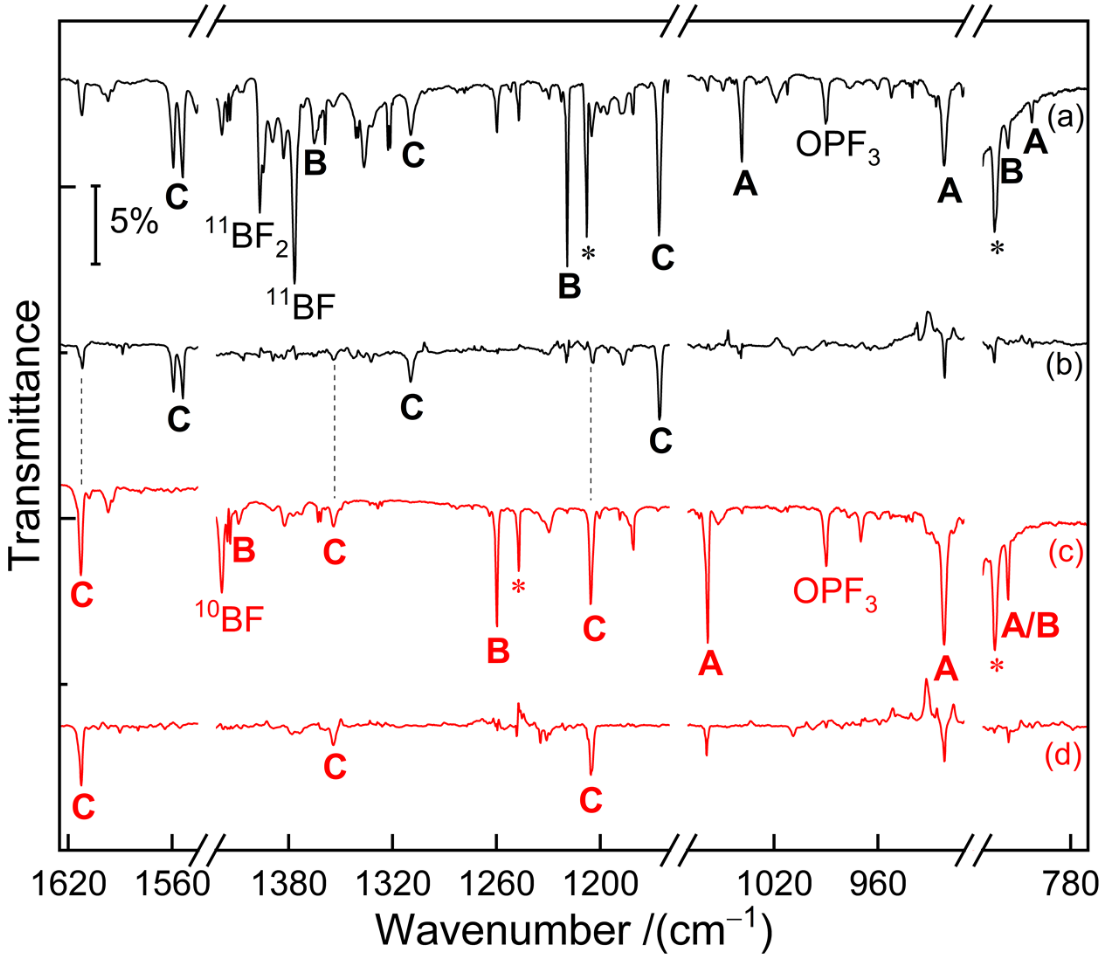

Figure S2. Infrared spectra obtained from codeposition of laser-ablated boron atoms in natural-abundance (a, b) and ^{10}B-enriched (c, d) with 0.05% PF_3 in solid neon. Spectra after 60 min of sample deposition at 5 K (a, c) and difference spectra observed after λ = 470 nm LED irradiation for 10 min (b, d). **A**: $B{=}PF_3$, **B**: $F_2B{-}PF$, **C**: $F_2B{-}P{=}BF$, unassigned bands are marked with asterisks.

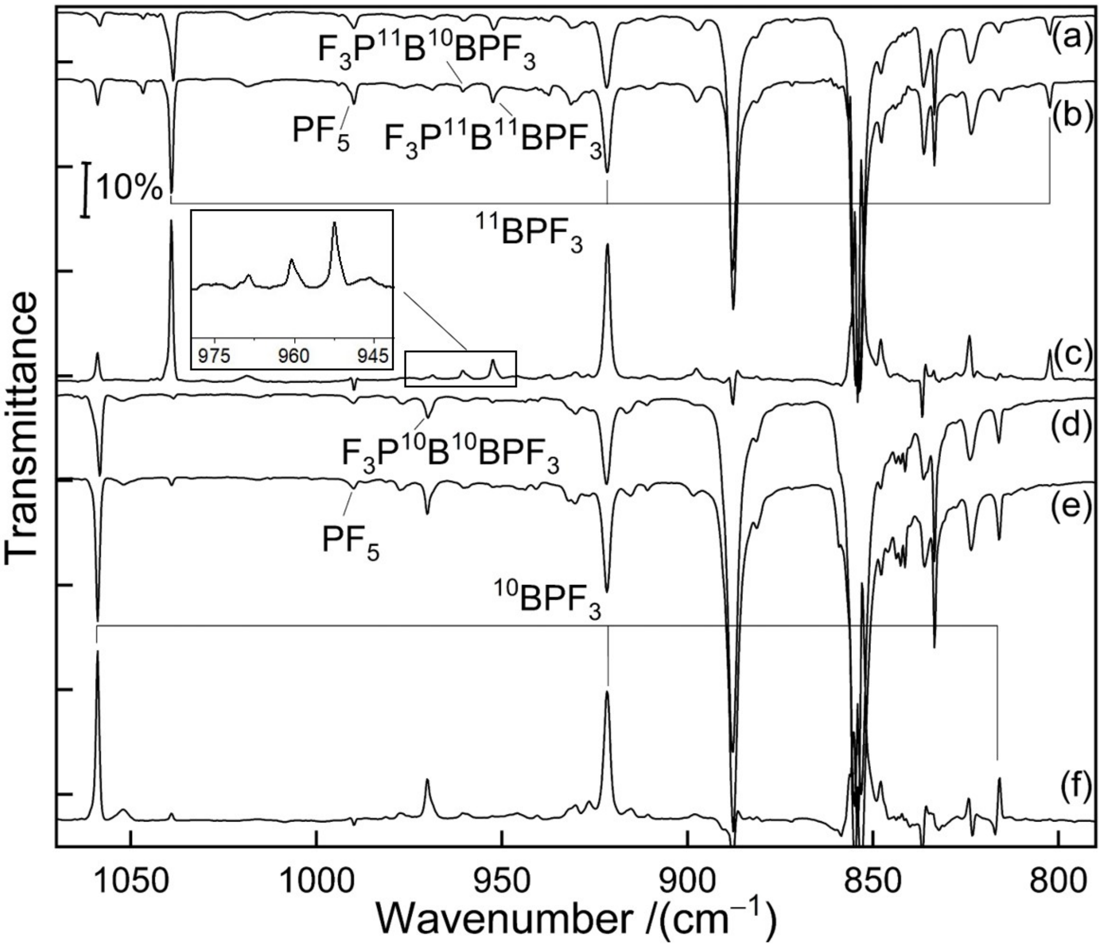

Figure S3. Infrared spectra obtained from codeposition of laser-ablated atoms in natural-abundance (a, b, c) and ^{10}B-enriched (d, e, f) with 0.05% PF_3 in neon (a, d), annealing to 9 K (b, e), and difference spectra observed after 15 min of $\lambda > 220$ nm irradiation (c, f).

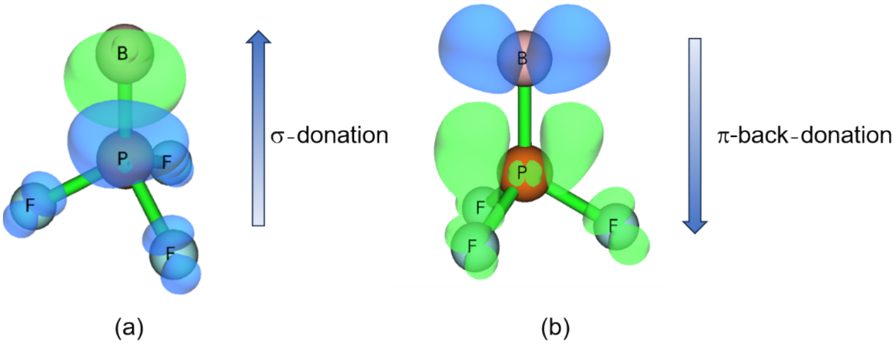

Figure S4. ETS-NOCV deformation maps for B=PF_3 (C_{3v}, $^4A_1$). Electron density flows from blue to green upon fragment combination.

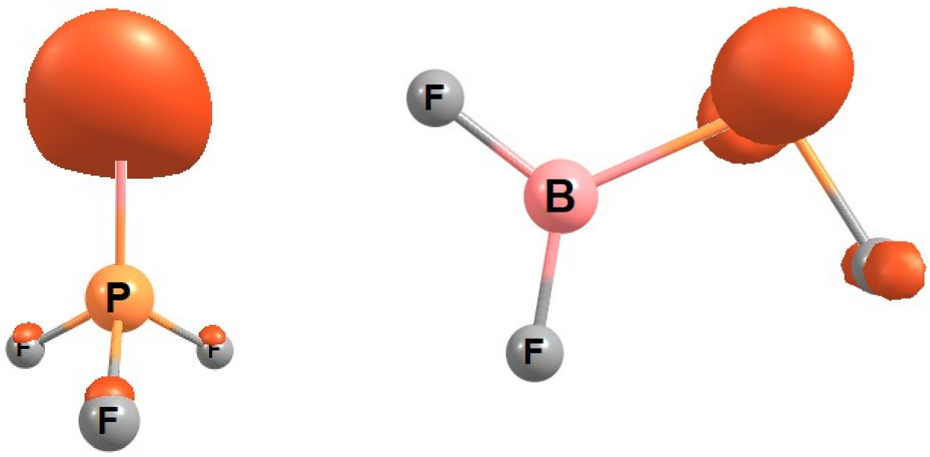

Figure S5. Spin density of B=PF_3 (C_{3v}, $^4A_1$) (left) and F_2B–PF (C_s, $^2A''$) (right) at 0.03 Å^{-3} calculated at B3LYP/aug-cc-pVTZ level.

Table S1. Calculated stretching wavenumbers ν (in cm^{-1}) and $^{10/11}B$ isotopic shifts (Δν, cm^{-1}) at the B3LYP/aug-cc-pVTZ level for $B{=}PF_3$ (**A**), $F_2B{-}PF$ (**B**) and $F_2B{-}P{=}BF$ (**C**) as well as IR intensities (in km mol^{-1}) in parentheses.

	$\nu(^{10}B)$	$\Delta\nu(^{11}B)$	stretching mode
$B{=}PF_3$	783.7 (84)	12.2	breathing
(C_{3v}, $^4A_1$)	875.9 (160×2)	0.0	antis. PF_3
	1044.8 (219)	21.9	B=P
$F_2B{-}PF$	629.5 (8)	5.4	B–P
(C_s, $^2A''$)	803.1 (114)	0.0	PF
	1250.7 (323)	40.0	sym. BF_2
	1426.4 (295)	49.3	antis. BF_2
$F_2B{-}P{=}BF$	626.0 (33)	5.0	B–P
(C_s, $^1A'$)	659.6 (2)	2.8	in-phase P=BF
	1201.3 (569)	38.4	sym. BF_2
	1361.3 (217)	46.5	antis. BF_2
	1631.1 (548)	57.6	out-of-phase P=BF

Table S2. Calculated stretching wavenumbers ν (in cm^{-1}) at the B3LYP/aug-cc-pVTZ level for $F_2B{-}P{=}BF$ (**C**) as well as IR intensities (in km mol^{-1}) in parentheses.

	$\nu(^{10}B/^{10}B)$	$\nu(^{10}B/^{11}B)$	$\nu(^{11}B/^{10}B)$	$\nu(^{11}B/^{11}B)$	stretching mode
$F_2B{-}P{=}BF$	626.0 (33)	620.8 (36)	625.9 (32)	620.6 (35)	B–P
(C_s, $^1A'$)	659.6 (2)	659.6 (2)	656.8 (1)	656.8 (1)	in-phase P=BF
	1201.3 (569)	1163.3 (520)	1200.8 (570)	1163.0 (521)	sym. BF_2
	1361.3 (217)	1315.0 (207)	1361.1 (213)	1314.8 (204)	antis. BF_2
	1631.1 (548)	1630.6 (547)	1574.1 (510)	1573.5 (509)	out-of-phase P=BF

Table S3. Observed and calculated stretching wavenumbers ν (in cm^{-1}) and $^{10/11}B$ isotopic shifts (Δν, cm^{-1}) at the B3LYP/aug-cc-pVTZ level for the antisymmetric P–B stretching mode of $F_3P{-}B{\equiv}B{-}PF_3$ (**D**) as well as IR intensities (in km mol^{-1}) in parentheses.

	obs. ν	cal. ν	obs. $\Delta\nu(^{11}B)$	calc $\Delta\nu(^{11}B)$
$F_3P{-}^{11}B{\equiv}^{11}B{-}PF_3$ (D_{3d}, $^1A_{1g}$)	952.5	940.5 (2075)		
$F_3P{-}^{11}B{\equiv}^{10}B{-}PF_3$ (D_{3d}, $^1A_{1g}$)	960.4	949.4 (2044)	7.9	8.9
$F_3P{-}^{10}B{\equiv}^{10}B{-}PF_3$ (D_{3d}, $^1A_{1g}$)	970.0	960.0 (2015)	9.6	10.6

Table S4 All calculated vibrational wavenumbers ν (in cm^{-1}) of $F_3P{-}^{11}B{\equiv}^{11}B{-}PF_3$ at the B3LYP/aug-cc-pVTZ level and the IR intensities (in km mol^{-1}) in parentheses.

	cal.
$F_3P{-}^{11}B{\equiv}^{11}B{-}PF_3$	3.0 (0)
(D_{3d}, $^1A_{1g}$)	29.9 (2·1)
	103.5 (2·0)
	220.9 (0)
	271.6 (2·0)
	305.2 (2·0)
	389.1 (2·0)
	393.7 (2·40)
	445.4 (296)
	535.6 (0)
	778.1 (57)
	906.7 (2·310)
	908.3 (2·0)
	918.3 (0)
	940.5 (2075)
	1693.0 (0)

Table S5 Energies and their differences for the $^4A_1$ and $^2A'$ states of $B{=}PF_3$ with different basis sets. All energies were calculated at the geometry optimized with the aug-cc-pVTZ basis set and include ZPE corrections at this level.

Basis set	E ($^4A_1$) / E_h	E ($^2A'$) / E_h	ΔE / kcal mol^{-1}
aug-cc-pVTZ	-664.8519085	-664.8545416	1.65
aug-cc-pVQZ	-664.9575655	-664.9578932	0.21
aug-cc-pV5Z	-664.9956937	-664.9947669	-0.58

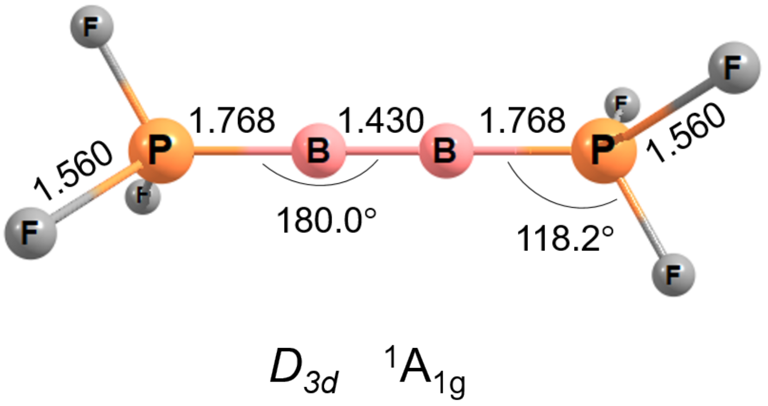

Figure S6. Optimized geometry of $F_3P{-}B{\equiv}B{-}PF_3$ at the B3LYP/aug-cc-pVTZ level of theory. Bond lengths (Å) and bond angles (°) are annotated.

Table S6. Calculated stretching wavenumbers ν (in cm^{-1}) and $^{10/11}$B isotopic shifts (Δν, cm^{-1}) at the B3LYP/aug-cc-pVTZ and CCSD(T)/aug-cc-pVTZ levels for B–PF_3 (C_s, ^{2}A'). IR intensities (in km mol^{-1}) in parentheses.

	cal. (B3LYP) ν(^{10}B)	cal. (CCSD(T)) ν(^{10}B)	cal. (B3LYP) Δν(^{11}B)	cal. (CCSD(T)) Δν(^{11}B)	mode
B–PF_3	345.3 (27)	362.7	7.5	8.4	BP stretch/PF_3 bend
(C_s, ^{2}A')	485.1 (18)	503.1	1.4	1.8	BP stretch/PF_3 bend
	800.5 (227)	850.0	0.1	0.1	PF stretch
	850.5 (231)	884.5	0.2	0.3	PF_2 symmetric stretch
	877.3 (156)	915.5	0.0	0.0	PF_2 antisymmetric stretch

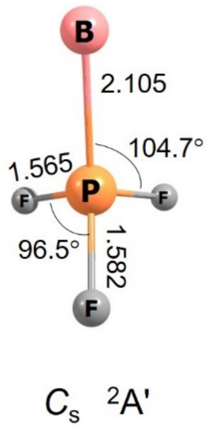

Figure S7. Optimized geometry of doublet B–PF_3 at the CCSD(T)/aug-cc-pVTZ level of theory. Bond lengths (Å) and bond angles (°) are annotated.

Table S7. Selected calculated properties of F_2B–P=BF at B3LYP and HF//CCSD(T) levels, both with aug-cc-pVTZ basis set.

property	B3LYP	HF//CCSD(T)
BPB angle	86.5°	83.3°
B···B distance	2.513 Å	2.445 Å
Wiberg B···B bond order in NAO basis	0.16	-
multi-center[11] BPB bond order in NAO basis	0.18	-
Mayer B···B bond order[12]	0.26	0.26
Mulliken B···B bond order[13]	0.12	0.12

Table S8. Orbital contributions to Mulliken bond order for the B=P bond of $F_2B{-}P{=}BF$ based on the Hartree-Fock wavefunction using the structure optimized at CCSD(T)/aug-cc-pVTZ level. Leading contributions are highlighted.

Orbital	Symmetry	Energy	Contributes
HOMO−15	A'	-1.71405	-0.02263053
HOMO−14	A'	-1.65166	0.00017158
HOMO−13	A'	-1.61806	-0.00449140
**HOMO−12**	A'	-0.90035	**0.27726266**
HOMO−11	A'	-0.85729	-0.02900871
HOMO−10	A'	-0.81639	-0.00083665
HOMO−9	A'	-0.76887	-0.00505582
HOMO−8	A'	-0.76880	0.00236880
HOMO−7	A"	-0.76397	-0.01793687
HOMO−6	A"	-0.71977	-0.00005339
HOMO−5	A'	-0.71420	0.13396779
HOMO−4	A"	-0.66633	-0.00009835
HOMO−3	A'	-0.65920	-0.00031986
HOMO−2	A'	-0.50454	**0.41667067**
**HOMO−1**	A'	-0.43368	0.05974367
**HOMO**	A"	-0.35270	**0.45568582**

Total Mulliken bond order: 1.26477285

Table S9. Orbital occupancy-perturbed Mayer bond order[1] for the B=P bond of F_2B–P=BF based on the Hartree-Fock wavefunction using the structure optimized at CCSD(T)/aug-cc-pVTZ level. Leading contributions are highlighted.

Orbital	Symmetry	Energy	Variance
HOMO−15	A'	-1.71405	0.022549
HOMO−14	A'	-1.65166	-0.006054
HOMO−13	A'	-1.61806	-0.000123
**HOMO−12**	A'	-0.90035	**-0.189162**
HOMO−11	A'	-0.85729	0.014529
HOMO−10	A'	-0.81639	-0.063331
HOMO−9	A'	-0.76887	-0.019265
HOMO−8	A'	-0.76880	-0.020841
HOMO−7	A"	-0.76397	-0.016175
HOMO−6	A"	-0.71977	-0.005656
HOMO−5	A'	-0.71420	-0.182590
HOMO−4	A"	-0.66633	-0.000377
HOMO−3	A'	-0.65920	-0.013569
**HOMO−2**	A'	-0.50454	**-0.514335**
HOMO−1	A'	-0.43368	-0.141084
**HOMO**	A"	-0.35270	**-0.652683**
Total Mayer bond order:	1.86922		

Table S10. Orbital contributions to Mulliken bond order for the P−B bond of F_2B−P=BF based on the Hartree-Fock wavefunction using the structure optimized at CCSD(T)/aug-cc-pVTZ level. Leading contributions are highlighted.

Orbital	Symmetry	Energy	Contributes
HOMO−15	A'	-1.71405	-0.00181459
HOMO−14	A'	-1.65166	0.00106377
HOMO−13	A'	-1.61806	0.00084860
**HOMO−12**	A'	-0.90035	**0.27315894**
HOMO−11	A'	-0.85729	0.05764208
HOMO−10	A'	-0.81639	0.08151648
HOMO−9	A'	-0.76887	-0.00008063
HOMO−8	A'	-0.76880	-0.00600820
HOMO−7	A"	-0.76397	0.01098869
HOMO−6	A"	-0.71977	-0.00126161
HOMO−5	A'	-0.71420	-0.10245818
HOMO−4	A"	-0.66633	-0.00066567
HOMO−3	A'	-0.65920	0.00666790
HOMO−2	A'	-0.50454	**0.24213504**
**HOMO−1**	A'	-0.43368	0.11381947
**HOMO**	A"	-0.35270	**0.19537098**

Total Mulliken bond order: 0.87518037

Table S11. Orbitals occupancy-perturbed Mayer bond order[1] for the P–B bond of F_2B–P=BF based on the Hartree-Fock wavefunction using the structure optimized at CCSD(T)/aug-cc-pVTZ level. Leading contributions are highlighted.

Orbital	Symmetry	Energy	Variance
HOMO−15	A'	-1.71405	-0.001394
HOMO−14	A'	-1.65166	-0.008582
HOMO−13	A'	-1.61806	-0.001618
**HOMO−12**	A'	-0.90035	**-0.289185**
HOMO−11	A'	-0.85729	-0.123285
HOMO−10	A'	-0.81639	-0.035805
HOMO−9	A'	-0.76887	-0.002634
HOMO−8	A'	-0.76880	-0.006593
HOMO−7	A"	-0.76397	-0.019961
HOMO−6	A"	-0.71977	-0.014623
HOMO−5	A'	-0.71420	0.011819
HOMO−4	A"	-0.66633	0.000127
HOMO−3	A'	-0.65920	-0.016436
**HOMO−2**	A'	-0.50454	**-0.352437**
HOMO−1	A'	-0.43368	-0.147845
**HOMO**	A"	-0.35270	**-0.225629**

Total Mayer bond order: 1.206548

Table S12. Orbital contributions to Mulliken bond order for the B···B interaction in $F_2B–P=BF$ based on the Hartree-Fock wavefunction using the structure optimized at CCSD(T)/aug-cc-pVTZ level. Leading contributions are highlighted.

Orbital	Symmetry	Energy	Contributes
HOMO−15	A'	-1.71405	0.00958452
HOMO−14	A'	-1.65166	0.00777727
HOMO−13	A'	-1.61806	-0.00555579
**HOMO−12**	A'	-0.90035	**0.09153395**
HOMO−11	A'	-0.85729	0.02833409
HOMO−10	A'	-0.81639	0.01580359
HOMO−9	A'	-0.76887	0.00164331
HOMO−8	A'	-0.76880	0.00484815
HOMO−7	A"	-0.76397	-0.03737224
HOMO−6	A"	-0.71977	0.00248406
HOMO−5	A'	-0.71420	-0.04458106
HOMO−4	A"	-0.66633	-0.00059706
HOMO−3	A'	-0.65920	0.00031735
HOMO−2	A'	-0.50454	-0.10990616
**HOMO−1**	A'	-0.43368	**0.10525781**
**HOMO**	A"	-0.35270	**0.05099353**

Total Mulliken bond order: 0.11922158

Table S13. Orbital occupancy-perturbed Mayer bond order[1] for the B···B interaction in F_2B–P=BF based on the Hartree-Fock wavefunction using the structure optimized at CCSD(T)/aug-cc-pVTZ level. Leading contributions are highlighted.

Orbital	Symmetry	Energy	Variance
HOMO−15	A'	-1.71405	-0.021041
**HOMO−14**	A'	-1.65166	**-0.050223**
HOMO−13	A'	-1.61806	0.002624
**HOMO−12**	A'	-0.90035	**-0.030174**
HOMO−11	A'	-0.85729	-0.000453
HOMO−10	A'	-0.81639	0.009016
HOMO−9	A'	-0.76887	-0.007897
HOMO−8	A'	-0.76880	0.002680
HOMO−7	A"	-0.76397	-0.006245
HOMO−6	A"	-0.71977	-0.007986
HOMO−5	A'	-0.71420	0.016624
HOMO−4	A"	-0.66633	-0.000373
HOMO−3	A'	-0.65920	-0.011383
HOMO−2	A'	-0.50454	0.168299
**HOMO−1**	A'	-0.43368	**-0.074272**
**HOMO**	A"	-0.35270	**-0.084940**

Total Mayer bond order: 0.259286

Table S14. Wiberg bond order decomposition in NAO basis for the B···B interaction in $F_2B–P=BF$ based on the Hartree-Fock wavefunction using the structure optimized at the CCSD(T)/aug-cc-pVTZ level.

Contri.	NAO type		NAO type
0.0022	Val(2s) s	---	Val(2s) s
0.0097	Val(2s) s	---	Val(2p) px
0.0270	Val(2s) s	---	Val(2p) py
0.0059	Val(2p) px	---	Val(2s) s
0.0030	Val(2p) px	---	Val(2p) px
0.0037	Val(2p) px	---	Val(2p) py
0.0023	Val(2p) py	---	Val(2s) s
0.0025	Val(2p) py	---	Val(2p) px
0.0315	Val(2p) py	---	Val(2p) py
0.0455	Val(2p) pz	---	Val(2p) pz

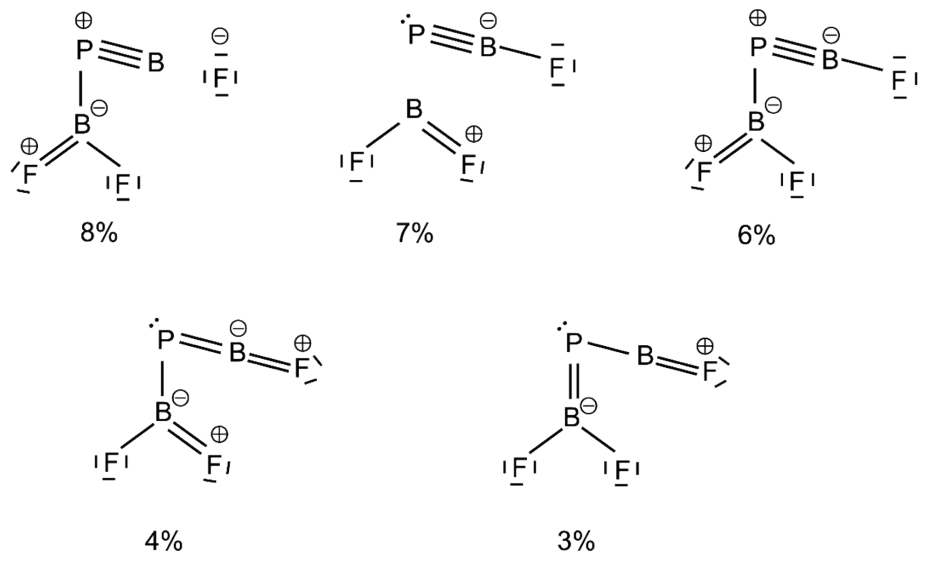

Figure S8 Further resonance structures with weights between 2 and 8% for $F_2B–P=BF$ at B3LYP/aug-cc-pVTZ level.

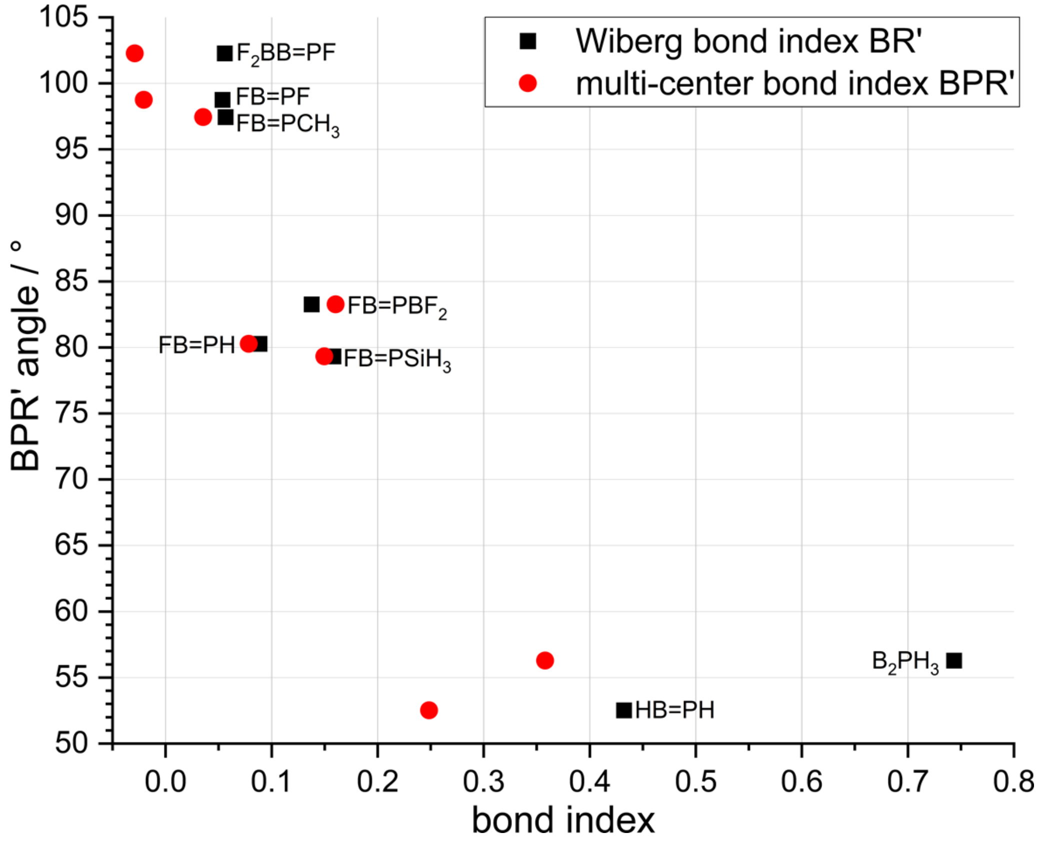

Figure S9. Correlation between the Wiberg bond index for the BR' interaction, the multi-center bond index[11] for the BPR' moiety (both in natural atomic orbital (NAO) basis) and the BPR' angle for different small phosphaborenes RB=PR', calculated at HF/aug-cc-pVTZ//CCSD(T)/aug-cc-pVTZ level.

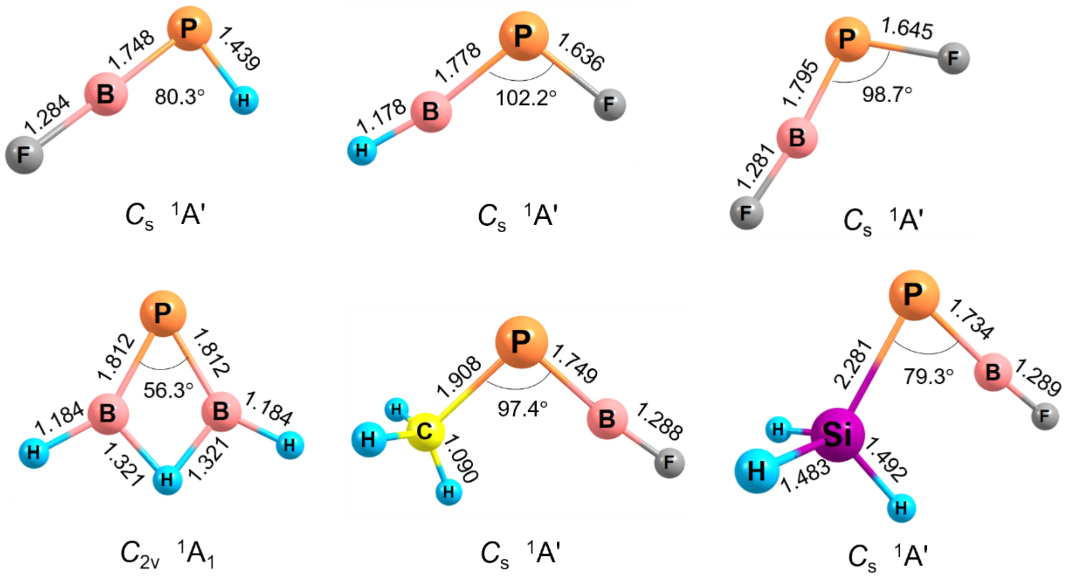

Figure S10. Optimized structures for different small phosphaborenes RB=PR' at the CCSD(T)/aug-cc-pVTZ level. Bond lengths (Å), bond angles (deg) and molecular symmetries are annotated.

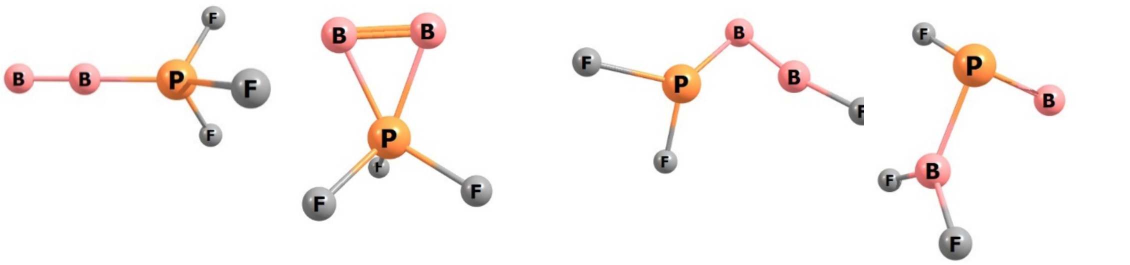

C_{3v}, $^1A_1$	C_s, $^1A'$	C_1, ^1A	C_1, ^3A
202.67	169.89	100.60	98.38

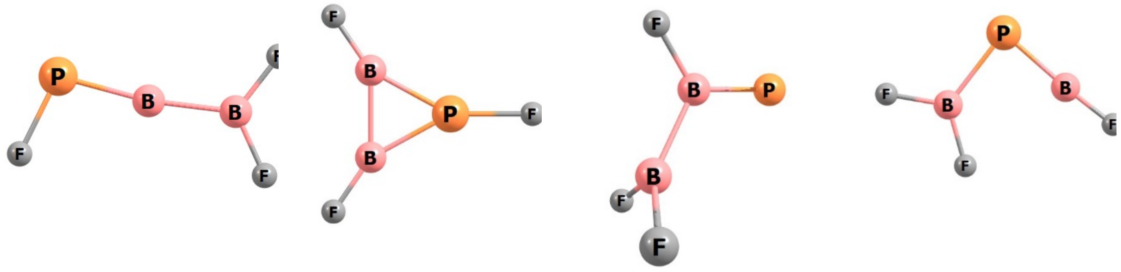

C_s, $^1A'$	C_{2v}, $^1A_1$	C_s, $^3A''$	C_s, $^1A'$
50.92	40.70	31.04	0

Figure S11. Isomers of the formula B_2PF_3 with relative stabilities (electronic energies + ZPE correction) in kcal mol^{-1} calculated at B3LYP/aug-cc-pVTZ level of theory.

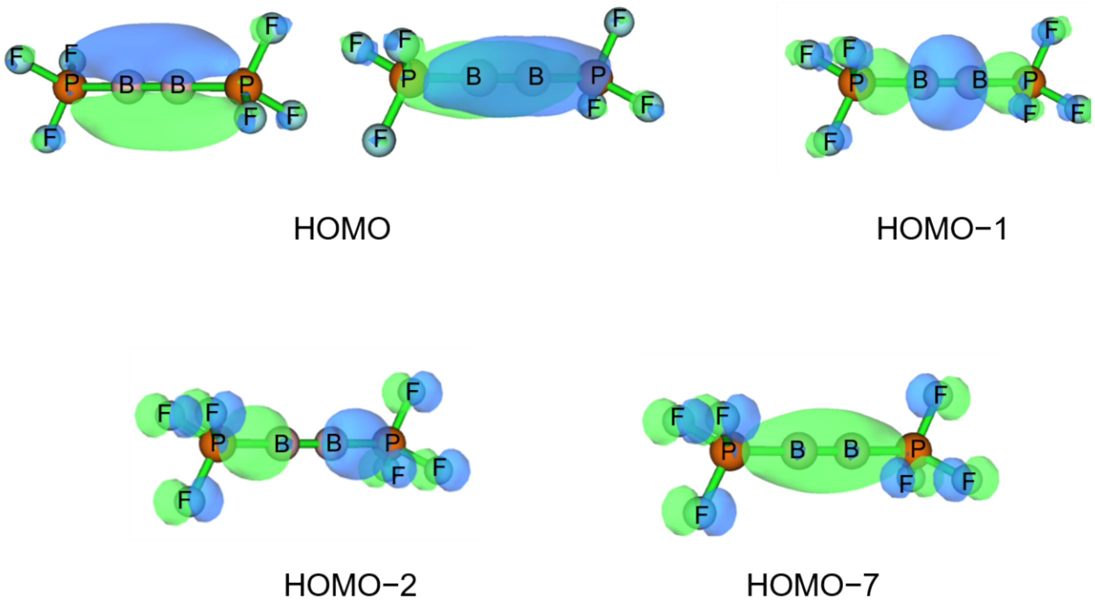

Figure S12. B–B and B–P bonding molecular orbitals of $F_3P{-}B{\equiv}B{-}PF_3$ calculated at B3LYP/aug-cc-pVTZ level of theory.

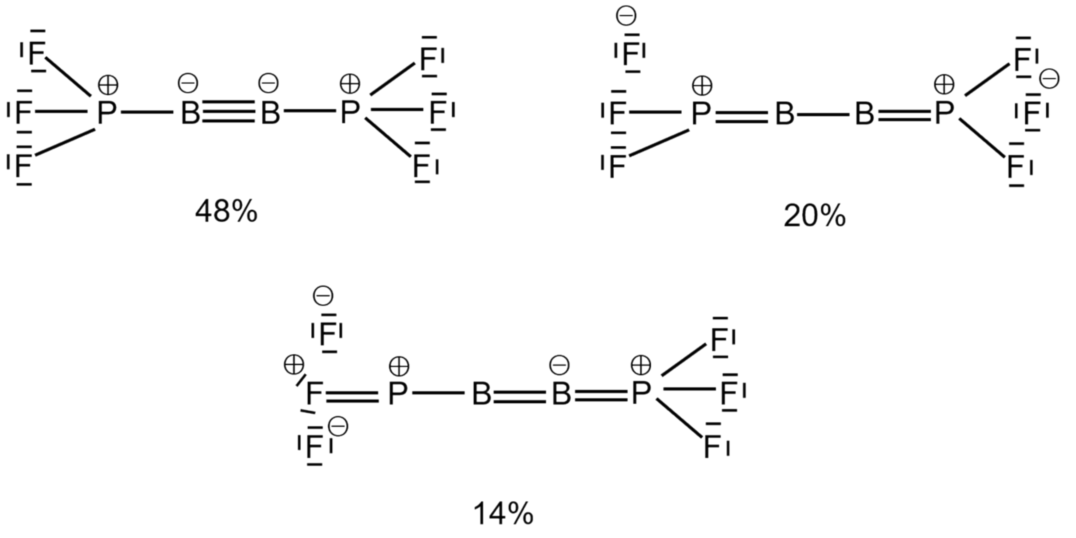

Figure S13. Leading resonance structure motifs for dimer $F_3P{-}B{\equiv}B{-}PF_3$ from natural resonance theory (NRT). The weights of structures with the same P–B–B–P bond orders, irrespective of the P–F fluorine bond orders, were added up.

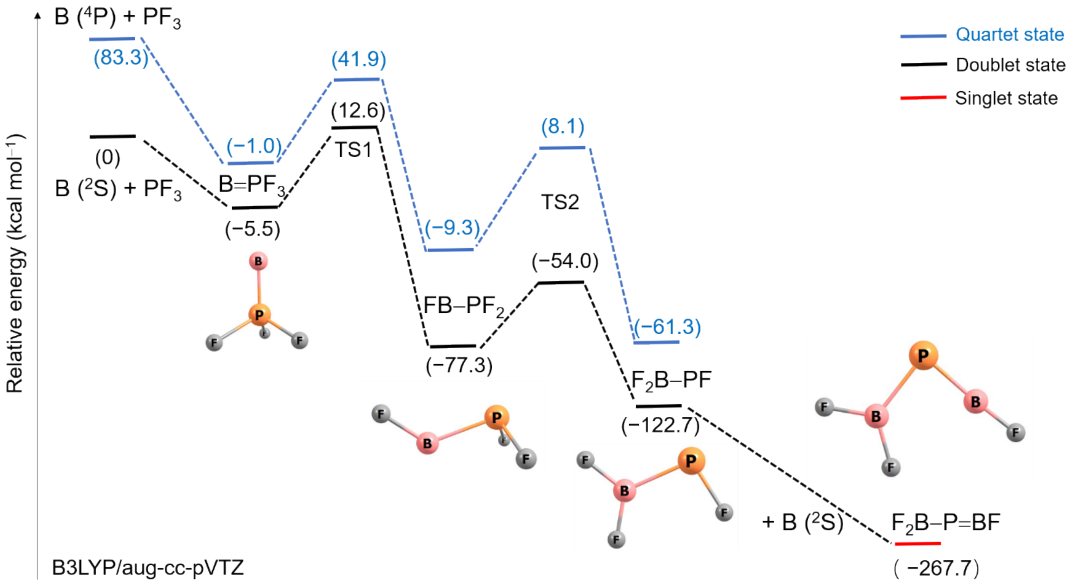

Scheme S1. Relative stabilities (electronic energies + ZPE correction) in kcal mol^{-1} for species formed from laser ablated boron atoms with PF_3 at the B3LYP/aug-cc-pVTZ level (distances not to scale).

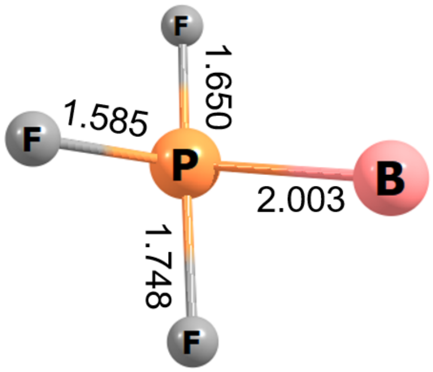

TS1-D (C_1, ^2A)

Frequency	Intensity
-204.6	–
113.2	1
207.0	6
311.0	33
420.6	6
514.3	49
528.9	49
712.4	332
858.7	153

Figure S14. Transition state structure (TS1-D) for the doublet hypersurface of Scheme S1 and its frequencies (cm^{-1}) calculated at the B3LYP/aug-cc-pVTZ level of theory.

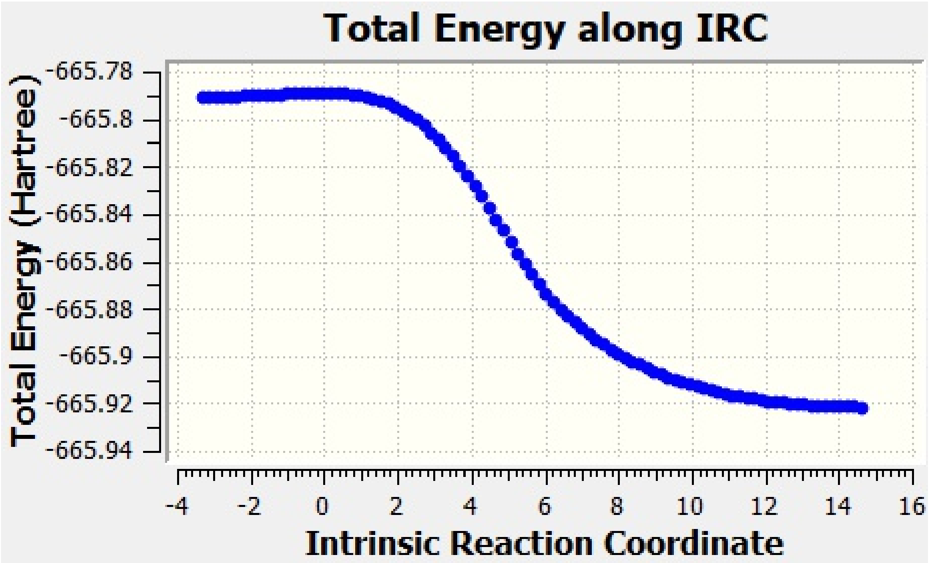

Figure S15. Intrinsic reaction coordinate (IRC) via the transition state TS1-D (Scheme S1) for the mutual isomerization of doublet B–PF_3 (C_s, $^2A'$) and doublet FB–PF_2 (C_1, ^2A) obtained at the B3LYP/aug-cc-pVTZ level of theory.

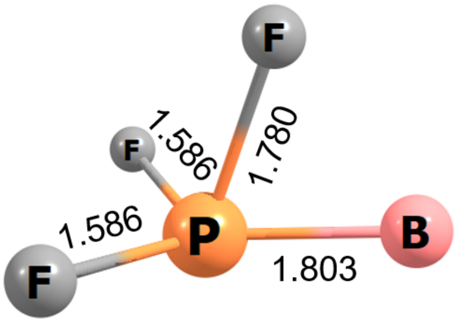

TS1-Q (C_s, $^4A''$)

Frequency	Intensity
-494.7	–
200.8	1
253.9	2
344.1	3
398.5	29
509.4	99
732.4	111
855.6	160
923.1	98

Figure S16. Transition state structure (TS1-Q) for the quartet hypersurface of Scheme S1 and its frequencies (cm^{-1}) calculated at the B3LYP/aug-cc-pVTZ level of theory.

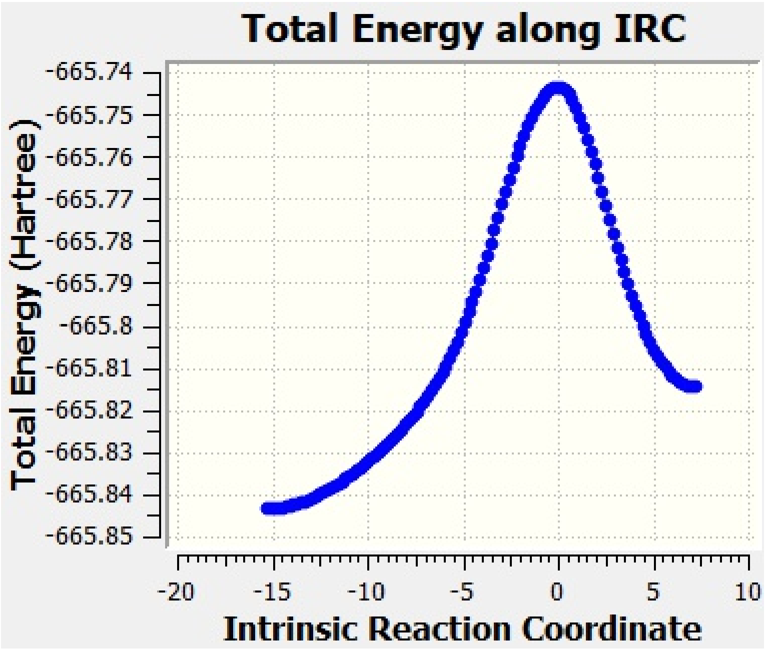

Figure S17. Intrinsic reaction coordinate (IRC) via the transition state TS1-Q (Scheme S1) for the mutual isomerization of quartet B=PF_3 (C_{3v}, $^4A_1$) and quartet FB–PF_2 obtained at the B3LYP/aug-cc-pVTZ level of theory.

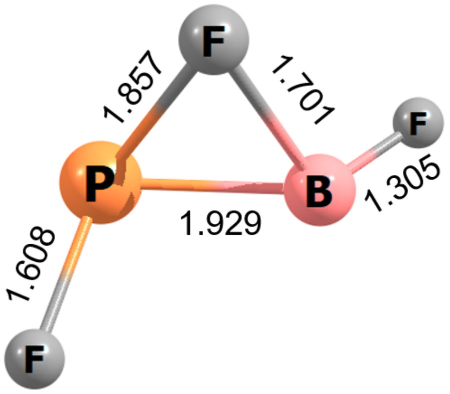

TS2-D (C_1, ^2A)

Frequency	Intensity
-375.7	–
117.5	9
222.2	4
298.1	3
385.7	2
557.7	79
619.4	58
802.0	153
1331.2	379

Figure S18. Transition state structure (TS2-D) for the doublet hypersurface of Scheme S1 and its frequencies (cm^{-1}) calculated at the B3LYP/aug-cc-pVTZ level of theory.

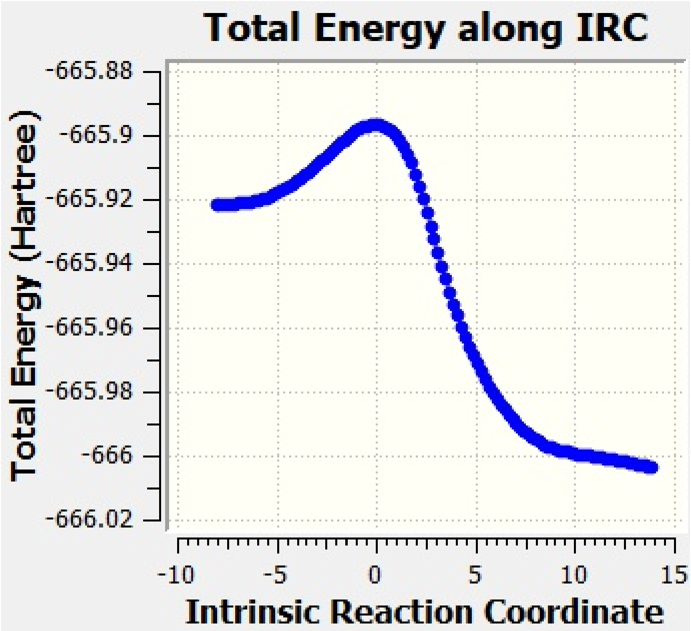

Figure S19. Intrinsic reaction coordinate (IRC) via the transition state TS2-D (Scheme S1) for the mutual isomerization of doublet FB–PF_2 and doublet F_2B–PF obtained at the B3LYP/aug-cc-pVTZ level of theory.

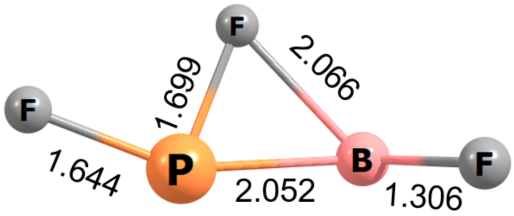

TS2-Q (C_1, ^4A)

Frequency	Intensity
-210.3	–
116.4	5
141.7	2
279.3	1
367.6	9
487.7	85
633.6	38
729.3	146
1312.9	235

Figure S20. Transition state structure (TS2-Q) for the quartet hypersurface of Scheme S1 and its frequencies (cm^{-1}) calculated at the B3LYP/aug-cc-pVTZ level of theory.

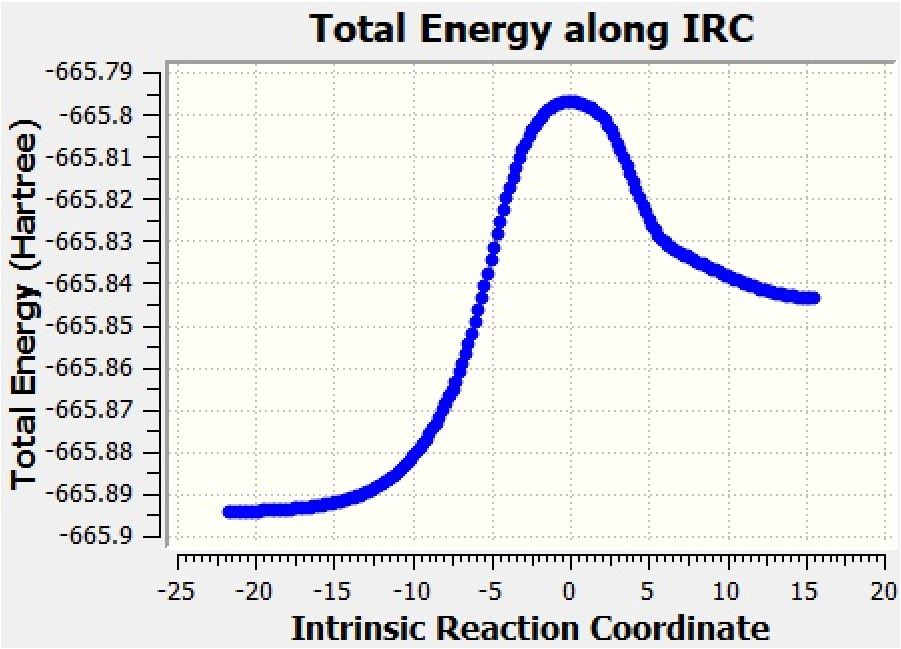

Figure S21. Intrinsic reaction coordinate (IRC) via the transition state TS2-Q (Scheme S1) for the mutual isomerization of quartet FB–PF_2 and quartet F_2B–PF obtained at the B3LYP/aug-cc-pVTZ level of theory.

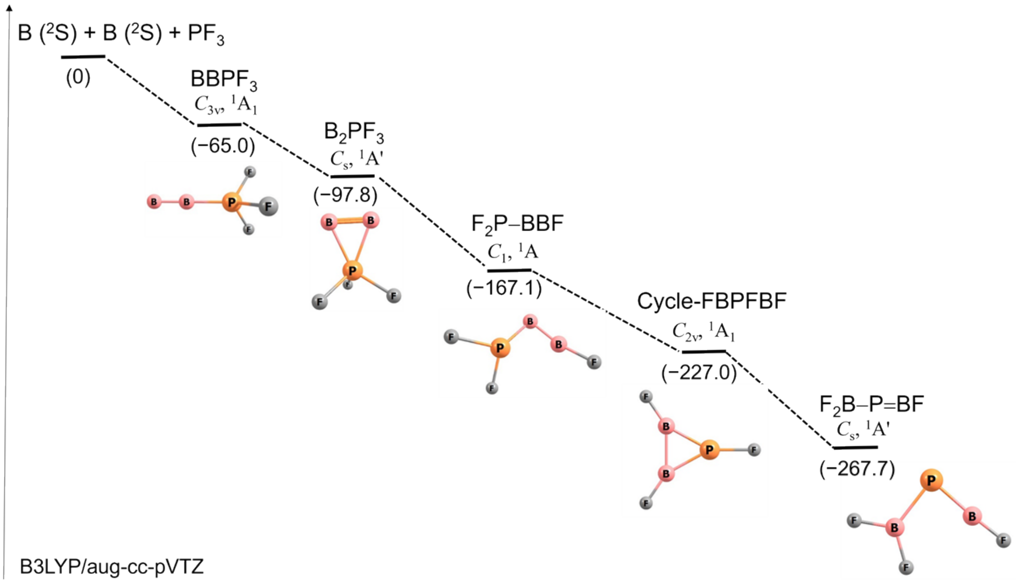

Scheme S2. Relative stabilities (electronic energies + ZPE correction) in kcal mol^{-1} for F_2B–P=BF species formed from laser ablated two boron atoms with PF_3 at the B3LYP/aug-cc-pVTZ level (distances not to scale).

Calculated atomic coordinates (in Å) of species for optimized structures at B3LYP/aug-cc-pVTZ level.

BPF_3 $^4A_1$ (C_{3v})

P	0.00000000	0.00000000	0.24969200
F	0.00000000	1.37392500	-0.51029900
F	-1.18985400	-0.68696200	-0.51029900
F	1.18985400	-0.68696200	-0.51029900
B	0.00000000	0.00000000	2.00653900

BPF_3 $^2A'$ (C_s)

B	2.10344100	0.93327600	0.00000000
P	0.24109500	-0.07352800	0.00000000
F	-0.52346800	-1.47676000	0.00000000
F	-0.52346800	0.54041000	1.23338600
F	-0.52346800	0.54041000	-1.23338600

F_2PBF ^2A (C_1)

F	0.31100690	0.80161472	1.33215440
F	0.31100690	0.80161472	-1.33215440
F	-0.02647023	-2.38272985	0.00000000
P	-0.51372484	0.50941220	0.00000000
B	0.46616201	-1.23311982	0.00000000

F_2PBF $^4A''$ (C_s)

F	0.31958700	-2.41283600	0.00000000
F	0.31958700	1.24948100	1.18076300
F	0.31958700	1.24948100	-1.18076300
P	-0.39452600	0.42039800	0.00000000
B	-0.54219200	-1.41622200	0.00000000

F_2BPF $^2A''$ (C_s)

F	1.283354126	1.119793743	0.000000000
F	-0.871231959	1.829134604	0.000000000
F	0.724901785	-1.761781536	0.000000000
B	-0.002281607	0.830587579	0.000000000
P	-0.680620345	-0.989597389	0.000000000

F_2BPF $^4A_2$ (C_{2v})

F	0.00000000	1.13835200	-1.79130900
F	0.00000000	-1.13835200	-1.79130900
F	0.00000000	0.00000000	2.63199100
B	0.00000000	0.00000000	-1.13466700
P	0.00000000	0.00000000	0.94859800

F_2BPBF $^1A'$ (C_s)

F	1.33402400	0.95651800	0.00000000
F	-0.49333500	2.27333900	0.00000000
F	1.13633100	-2.28277600	0.00000000

B	0.00000000	1.04226900	0.00000000
P	-1.23935000	-0.42704300	0.00000000
B	0.15941500	-1.46588700	0.00000000

TS1-D ^{2}A (C_1)

P	0.07552200	-0.10199600	-0.26439500
F	-1.64999800	0.16179700	-0.34985200
F	1.70040200	-0.38140700	-0.19312400
F	0.22913900	1.26465700	0.52375000
B	-0.72974500	-1.57509800	0.82779200

TS1-Q ^{4}A" (C_s)

P	-0.44531400	-0.08750000	0.00000000
F	1.28205400	-0.51695400	0.00000000
F	-0.21125100	0.85234300	1.25648800
F	-0.21125100	0.85234300	-1.25648800
B	-0.21125100	-1.87541500	0.00000000

TS2-D ^{2}A (C_1)

F	1.81714600	-0.50006200	-0.50740300
F	-0.40471000	1.21774200	-0.28032900
F	-2.15009400	-0.53999100	0.02948500
P	0.74389400	0.02908400	0.56706700
B	-0.90389600	-0.40709100	-0.33635400

TS2-Q ^{4}A (C_1)

F	-2.33674200	-0.20375500	-0.34739100
F	2.09559100	-0.12452800	-0.22932400
F	0.14419400	1.16663200	0.02614500
P	0.53336200	-0.48348100	0.13780300
B	-1.42556300	-0.05858600	0.57761900

F_3PBBPF_3 $^1A_{1g}$ (D_{3d})

P	0.00000000	0.00000000	2.48280100
F	0.00000000	1.37540800	3.21976800
F	1.19113900	-0.68770400	3.21976800
F	-1.19113900	-0.68770400	3.21976800
B	0.00000000	0.00000000	0.71490900
P	0.00000000	0.00000000	-2.48280100
F	-1.19113900	0.68770400	-3.21976800
F	0.00000000	-1.37540800	-3.21976800
F	1.19113900	0.68770400	-3.21976800
B	0.00000000	0.00000000	-0.71490900

Calculated atomic coordinates (in Å) of species for optimized structures at CCSD(T)/aug-cc-pVTZ level.

BPF_3 $^4A_1$ (C_{3v})

P	-0.000008263	0.000000000	-0.235793905
F	1.365090707	0.000000000	0.514615238
F	-0.682537079	-1.182190443	0.514595530
F	-0.682537079	1.182190443	0.514595530
B	-0.000008286	0.000000000	-1.998128599

BPF_3 $^2A'$ (C_s)

B	2.099889777	0.913607960	0.000000000
P	0.236597227	-0.064726255	0.000000000
F	-0.500472577	-1.464542222	0.000000000
F	-0.530941213	0.539734258	1.222213980
F	-0.530941213	0.539734258	-1.222213980

F_2PBF ^2A (C_1)

F	-0.769760422	1.335360351	-0.316191135
F	-1.438081167	-1.002822946	-0.320083268
F	2.378735456	-0.070126500	0.022337676
P	-0.508092766	-0.012568756	0.520449189
B	1.223511899	-0.439383149	-0.454077461

F_2PBF $^4A''$ (C_s)

F	0.329413417	-2.401066767	0.000000000
F	0.321339691	1.241846469	1.165977190
F	0.321339691	1.241846469	-1.165977190
P	-0.395584769	0.424415172	0.000000000
B	-0.554465030	-1.416739343	0.000000000

F_2BPF $^2A''$ (C_s)

F	-1.047088012	1.338529181	-0.000028966
F	-1.875622712	-0.775827646	-0.000000428
F	1.795483922	0.642823494	0.000003156
B	-0.830569276	0.038062301	-0.000006542
P	0.954284078	-0.737473330	0.000017779

F_2BPF $^4A_2$ (C_{2v})

F	0.000000000	-1.137759285	1.782569742
F	0.000000000	1.137759285	1.782569742
F	0.000000000	0.000000000	-2.610781676
B	0.000000000	0.000000000	1.123007830
P	0.000000000	0.000000000	-0.942187278

F_2BPBF $^1A'$ (C_s)

F	1.326575047	0.886162081	0.000000000
F	-0.459718333	2.258017295	0.000000000
F	1.169454279	-2.201833692	0.000000000
B	-0.007634862	1.011588587	0.000000000

P	-1.284441205	-0.428991901	0.000000000
B	0.152850074	-1.428522369	0.000000000

FBPH [1]A' (C_s)

P	-0.106262398	-1.218741190	0.000000000
B	-0.004278009	0.526228466	0.000000000
H	1.323443088	-1.058800760	0.000000000
F	0.038297319	1.809716483	0.000000000

HBPF [1]A' (C_s)

P	-0.068763595	-1.093794200	0.000000000
B	-0.017108476	0.683178238	0.000000000
H	-0.186278844	1.848888661	0.000000000
F	1.519896535	-1.484006388	0.000000000

FBPF [1]A' (C_s)

F	-0.330121324	2.195624319	0.000000000
P	0.657062866	-0.711430516	0.000000000
B	-0.008145425	0.956041352	0.000000000
F	-0.759839116	-1.546188156	0.000000000

H_2BPBH [1]A_1 (C_{2v})

B	-0.854861196	-0.775382696	0.000000000
P	0.000003719	0.822401911	0.000000000
B	0.854861480	-0.775386265	0.000000000
H	1.903797054	-1.323741234	0.000000000
H	-1.903799680	-1.323732048	0.000000000
H	-0.000002376	-1.781989668	0.000000000

CH_3PBF [1]A' (C_s)

B	0.960620542	-0.038234764	-0.000078580
P	-0.621171727	-0.784185087	0.000068358
C	-1.651770909	0.821524335	0.000123999
H	-2.283167555	0.807784499	0.888551872
H	-2.283343335	0.807730070	-0.888178149
H	-1.061829139	1.736310367	0.000036935
F	2.172463122	0.396767580	-0.000189436

CH_3PBF [1]A' (C_s)

B	-1.145878939	0.036890237	0.000000325
P	0.076015537	1.267276195	0.000000248
Si	1.368933092	-0.611848666	0.000000481
H	2.226873974	-0.614286363	1.209647015
H	2.226872041	-0.614288567	-1.209647387
H	0.567435149	-1.869797013	-0.000000148
F	-2.111002854	-0.817557822	0.000000466

References

[1] S. I. Gorelsky, *J. Chem. Theory Comput.* **2012**, *8*, 908.

[2] T. Schlöder, T. Vent-Schmidt, S. Riedel, *Angew. Chem. Int. Ed.* **2012**, *51*, 12063.

[3] M. J. Frisch, G. W. Trucks, H. B. Schlegel, G. E. Scuseria, M. A. Robb, J. R. Cheeseman, G. Scalmani, V. Barone, G. A. Petersson, H. Nakatsuji, X. Li, M. Caricato, A. V. Marenich, J. Bloino, B. G. Janesko, R. Gomperts, B. Mennucci, H. P. Hratchian, J. V. Ortiz, A. F. Izmaylov, J. L. Sonnenberg, D. Williams-Young, F. Ding, F. Lipparini, F. Egidi, J. Goings, B. Peng, A. Petrone, T. Henderson, D. Ranasinghe, V. G. Zakrzewski, J. Gao, N. Rega, G. Zheng, W. Liang, M. Hada, M. Ehara, K. Toyota, R. Fukuda, J. Hasegawa, M. Ishida, T. Nakajima, Y. Honda, O. Kitao, H. Nakai, T. Vreven, K. Throssell, J. A. Montgomery, Jr., J. E. Peralta, F. Ogliaro, M. J. Bearpark, J. J. Heyd, E. N. Brothers, K. N. Kudin, V. N. Staroverov, T. A. Keith, R. Kobayashi, J. Normand, K. Raghavachari, A. P. Rendell, J. C. Burant, S. S. Iyengar, J. Tomasi, M. Cossi, J. M. Millam, M. Klene, C. Adamo, R. Cammi, J. W. Ochterski, R. L. Martin, K. Morokuma, O. Farkas, J. B. Foresman, and D. J. Fox, *Gaussian 16*, Gaussian, Inc., Wallingford CT, **2016**.

[4] a) P. J. Stephens, F. J. Devlin, C. F. Chabalowski, M. J. Frisch, *J. Phys. Chem.* **1994**, *98*, 11623; b) A. D. Becke, *J. Chem. Phys.* **1993**, *98*, 5648; c) C. Lee, W. Yang, R. G. Parr, *Phys. Rev. B* **1988**, *37*, 785; d) S. H. Vosko, L. Wilk, M. Nusair, *Can. J. Phys.* **1980**, *58*, 1200.

[5] a) K. Raghavachari, G. W. Trucks, J. A. Pople, M. Head-Gordon, *Chem. Phys. Lett.* **1989**, *157*, 479; b) G. D. Purvis, R. J. Bartlett, *J. Chem. Phys.* **1982**, *76*, 1910.

[6] H.-J. Werner, P. J. Knowles, G. Knizia, F. R. Manby, M. Schütz, P. Celani, W. Györffy, D. Kats, T. Korona, R. Lindh, A. Mitrushenkov, G. Rauhut, K. R. Shamasundar, T. B. Adler, R. D. Amos, S. J. Bennie, A. Bernhardsson, A. Berning, D. L. Cooper, M. J. O. Deegan, A. J. Dobbyn, F. Eckert, E. Goll, C. Hampel, A. Hesselmann, G. Hetzer, T. Hrenar, G. Jansen, C. Köppl, S. J. R. Lee, Y. Liu, A. W. Lloyd, Q. Ma, R. A. Mata, A. J. May, S. J. McNicholas, W. Meyer, T. F. Miller III, M. E. Mura, A. Nicklass, D. P. O'Neill, P. Palmieri, D. Peng, K. Pflüger, R. Pitzer, M. Reiher, T. Shiozaki, H. Stoll, A. J. Stone, R. Tarroni, T. Thorsteinsson, M. Wang, M. Welborn, *MOLPRO, version 2019.2, a package of ab initio programs*.

[7] R. A. Kendall, T. H. Dunning, R. J. Harrison, *J. Chem. Phys.* **1992**, *96*, 6796.

[8] E. D. Glendening, J. K. Badenhoop, A. E. Reed, J. E. Carpenter, J. A. Bohmann, C. M. Morales, P. Karafiloglou, C. R. Landis, F. Weinhold, *NBO 7.0*, Theoretical Chemistry Institute, University of Wisconsin, Madison, WI, **2018**.

[9] T. Lu, F. Chen, *J. Comput. Chem.* **2012**, *33*, 580.

[10] G. A. Zhurko, *Chemcraft - graphical program for visualization of quantum chemistry computations. https://www.chemcraftprog.com*, Ivanovo (Russia), **2005**.

[11] M. Giambiagi, M. S. de Giambiagi, K. C. Mundim, *Struct. Chem.* **1990**, *1*, 423.

[12] A. J. Bridgeman, G. Cavigliasso, L. R. Ireland, J. Rothery, *J. Chem. Soc., Dalton Trans.* **2001**, 2095.

[13] R. S. Mulliken, *J. Chem. Phys.* **1955**, *23*, 1833.
